# Histone Chaperone NAP1 Mediates Sister Chromatid Resolution by Counteracting Protein Phosphatase 2A

**DOI:** 10.1371/journal.pgen.1003719

**Published:** 2013-09-26

**Authors:** Yuri M. Moshkin, Cecile M. Doyen, Tsung-Wai Kan, Gillian E. Chalkley, Karen Sap, Karel Bezstarosti, Jeroen A. Demmers, Zeliha Ozgur, Wilfred F. J. van Ijcken, C. Peter Verrijzer

**Affiliations:** 1 Department of Biochemistry and Centre for Biomedical Genetics, Erasmus University Medical Center, Rotterdam, The Netherlands; 2 Proteomics Center, Erasmus University Medical Center, Rotterdam, The Netherlands; 3 Genomics Centre, Erasmus University Medical Center, Rotterdam, The Netherlands; Duke University, United States of America

## Abstract

Chromosome duplication and transmission into daughter cells requires the precisely orchestrated binding and release of cohesin. We found that the *Drosophila* histone chaperone NAP1 is required for cohesin release and sister chromatid resolution during mitosis. Genome-wide surveys revealed that NAP1 and cohesin co-localize at multiple genomic loci. Proteomic and biochemical analysis established that NAP1 associates with the full cohesin complex, but it also forms a separate complex with the cohesin subunit stromalin (SA). NAP1 binding to cohesin is cell-cycle regulated and increases during G2/M phase. This causes the dissociation of protein phosphatase 2A (PP2A) from cohesin, increased phosphorylation of SA and cohesin removal in early mitosis. PP2A depletion led to a loss of centromeric cohesion. The distinct mitotic phenotypes caused by the loss of either PP2A or NAP1, were both rescued by their concomitant depletion. We conclude that the balanced antagonism between NAP1 and PP2A controls cohesin dissociation during mitosis.

## Author Summary

Eukaryotic DNA is assembled into a nucleo-protein structure called chromatin. Nucleosomes are the basic building blocks of chromatin, comprising 147 bp of DNA tightly wrapped around a histone protein core. Histone chaperones mediate nucleosome assembly by preventing non-productive aggregation of histones with DNA. Here, we describe an unexpected function for the canonical histone chaperone NAP1 in sister chromatid resolution. The precisely orchestrated binding and release of cohesin is crucial for proper chromosome segregation in mitosis. Cohesin holds newly replicated sister chromatids together till early mitosis. Then, it is first removed from the chromosome arms and lastly from the centromeres. This process ensures proper chromosome resolution and segregation into daughter cells. Cohesin removal from the arms is initiated by mitotic kinases in early mitosis, but can be counteracted by protein phosphatase 2A (PP2A). We found that NAP1 blocks protein phosphatase 2A binding to chromosomal cohesin, thereby allowing cohesin phosphorylation and dissociation from the chromosome arms. Functional *in vivo* experiments established that the antagonistic activities of NAP1 and PP2A control the chromosomal cohesin cycle and sister chromatid resolution. These results provide a novel chromatin-assembly-independent mitotic function for a histone chaperone.

## Introduction

Histone chaperones perform crucial functions during the duplication of eukaryotic genomes [1]–[2]. They guide the posttranslational processing and trafficking of newly-synthesized histones to replication forks and mediate replication-coupled chromatin assembly [2]–[8]. Histone chaperones CAF1, ASF1 and HIRA bind histone H3/H4 tetramers, whereas NAP1 binds both H3/H4 tetramers and H2A/H2B dimers. Although originally identified as factors that prevent aggregation and direct the assembly of histones on DNA [9], it turned out that histone chaperones play a variety of regulatory roles in chromosome biology. In addition to replication-coupled chromatin assembly, histone chaperones function in gene-specific transcription control, DNA repair and direct specific histone modifications [10]–[15].

Histone chaperones achieve these diverse functions through cooperation with other factors, such as histone modifying enzymes and ATP-dependent chromatin remodelers [15]–[20]. For example, ASF1 and NAP1 cooperates with histone modifying factors to differentially modulate local chromatin during NOTCH signaling [15], [21]. NAP1 associates with RLAF (RPD3 and LID associated factors), an assemblage of the histone deacetylase RPD3, histone H3 lysine 4 demethylase LID/KDM5, SIN3A, PF1, EMSY and MRG15. NAP1 recruits RLAF to the *(E)Spl* NOTCH-regulated genes to generate a repressive chromatin structure and mediate transcriptional silencing [15].

A specific function for histone chaperones during mitosis has not been established. Suggestively, we noted the potential association between NAP1 and cohesin in a proteomic survey of histone chaperones [15]. Cohesin is the conserved protein complex that mediates cohesion between sister chromatids after replication, which is crucial for proper chromosome segregation in mitosis and meiosis. The core of cohesin is formed by Stromalin (SA/SCC3), and a tripartite ring comprising SMC1, SMC3 and RAD21/SCC1. The cohesin ring embraces and holds sister chromatids together [22]–[23]. For a comprehensive discussion of mitotic cohesin dynamics we refer to a number of excellent reviews [24]–[31].

Briefly, cohesin binds chromosomes prior to DNA replication, enabling the linkage of newly replicated sister chromatids from S- through G2 phase. By metaphase, juxtaposed chromatids are only connected at their centromeric regions and have separate chromosome arms. This process is referred to as sister chromatid resolution and requires cohesin release from the arms, but not from the centromeres. During prophase, Polo-like kinase and potentially other mitotic kinases, phosphorylate SA, which triggers the bulk dissociation of cohesin from the chromosome arms [32]–[34]. This step also requires the cohesin releasing complex WAPL-PDS5 that interacts transiently with cohesin at mitotic entry [35]–[38]. All this time, centromeric cohesin remains associated and is protected from phosphorylation by the Shugoshin(Sgo)/MeiS332 family of proteins, which act in conjunction with PP2A [39]–[45]. At anaphase, separase-mediated cleavage of RAD21 causes the dissociation of centromeric cohesin, allowing sister chromatid segregation [46]–[47].

Motivated by the potential interaction between NAP1 and cohesin [15], we wondered if NAP1 might function in the cohesin chromosome binding and release cycle. We established that NAP1 and cohesin interact functionally. Through counteracting PP2A access to cohesin in early mitosis, NAP1 is a crucial regulator of the chromosomal cohesin cycle. Loss of NAP1 severely compromised cohesin release from the chromosome arms and sister chromatid resolution. These results uncover a mitotic function for NAP1 that is separate from its role in nucleosome assembly.

## Results

### NAP1 is required for sister chromatid resolution

**Figure 1:**
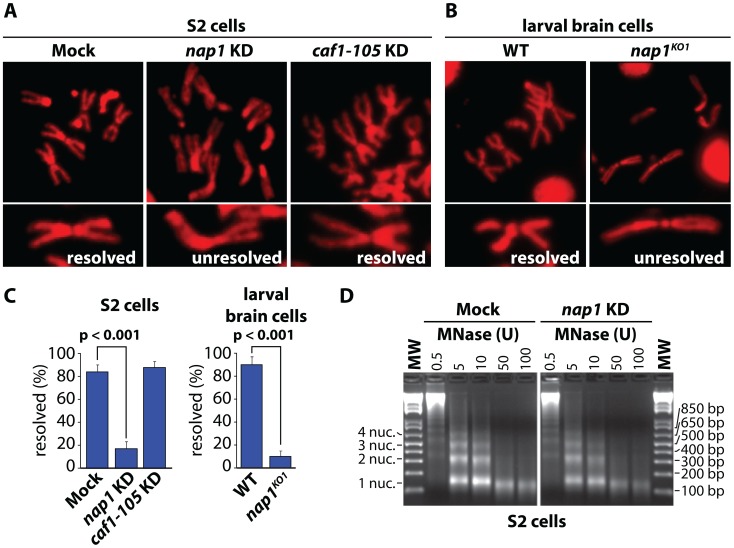
NAP1 is required for sister chromatid resolution. (**A**) Analysis of mitotic chromosomes from colchicine-treated S2 cells stained with DAPI (shown in red). Sister chromatids from cells depleted for NAP1 (KD) frequently do not resolve properly, compared to those from mock-treated or CAF1-105 depleted cells. Representative mitotic chromosomes are shown at higher magnification. (**B**) Mitotic chromosomes from wild type (WT) or *nap1^KO1^* larvae brain cells. (**C**) Quantification of sister chromatid resolution in the presence or absence of NAP1. The frequency of mitotic cells with resolved sister chromatids is significantly lower in NAP1 depleted cells than in mock-treated or CAF1-105 depleted cells. Likewise, sister chromatid resolution was significantly compromised in brain cells from *nap1^KO1^* compared to wild type larvae. Each analysis was based on >30 cells from 3 biological replicates and significance was determined by a χ^2^-test. (**D**) NAP1 knockdown does not affect nucleosome spacing or MNase sensitivity. Formaldehyde cross-linked chromatin prepared from mock-treated or NAP1 depleted S2 cells was digested with indicated units of MNase. Purified DNA was loaded onto a 2% agarose gel and stained with ethidium bromide. DNA fragments corresponding to mono-, di-, tri- and tetranucleosomes are indicated.

To examine the role of NAP1, we analyzed mitotic chromosomes prepared from colchicine-treated S2 cells after RNAi-mediated depletion of NAP1. Loss of NAP1, but not loss of the histone chaperone CAF1, caused a striking increase in the number of unresolved sister chromatids (Figure 1A and Figure S1A). In addition, depletion of NAP1 caused reduced cell proliferation, mitotic defects, an accumulation of poly/aneuploid cells and an increased portion of cells in the G1-phase of the cell cycle (Figure S1B–D). We also analyzed mitotic chromosomes from *Drosophila* larvae homozygous for the NAP1 knockout allele *nap1^KO1^* [48]. *Nap1^KO1^* homozygous flies are semi-lethal, and male escapers are sterile. Immunoblotting confirmed that NAP1 was not detectable in larvae homozygous for *nap1^KO1^*, whereas cohesin levels were unaffected (Figure S1A). We prepared mitotic chromosomes from colchicine-treated larval brain cells. In cells lacking NAP1 we observed a dramatic increase in cohesively linked sister chromatids, compared to wild type cells (Figure 1B). Quantification of S2 cells and larval brain cells with either resolved or unresolved sister chromatids confirmed the crucial role of NAP1 in this process (Figure 1C). However, we observed no changes in mitotic chromosome morphology, immunostaining efficiency of histone H3, micrococcal nuclease (MNase) sensitivity or nucleosome spacing upon depletion of NAP1 (Figure 1D and Figure S1E). This suggests that there are no gross changes in chromatin organization due to loss of NAP1. We conclude that NAP1 is required for sister chromatid resolution and normal mitosis.

### Cohesin release is compromised in cells depleted for NAP1

**Figure 2:**
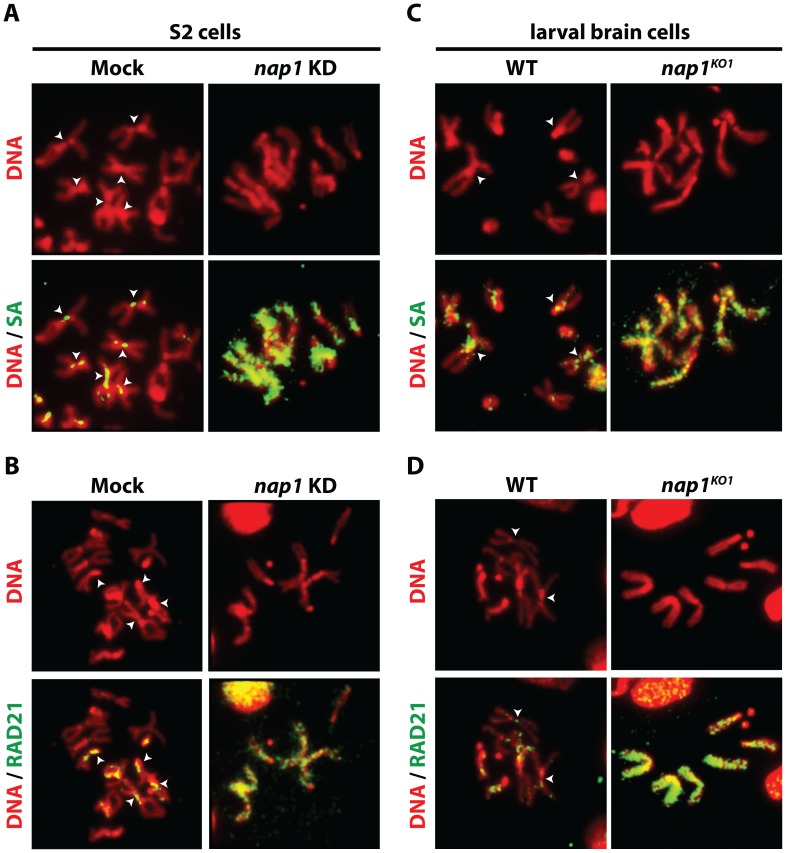
Loss of NAP1 compromises cohesin removal from mitotic chromosome arms. (**A**) Indirect immunofluorescent analysis of SA (green) binding to mitotic chromosomes from mock-treated or NAP1 knockdown S2 cells. DNA visualized by DAPI is shown in red. The centromeric localization of SA in mock-treated cells is indicated by arrowheads. Upon NAP1 KD, there is a dramatic accumulation of SA on the mitotic chromosome arms in ∼80% of cells. (**B**) Indirect immunofluorescent analysis of RAD21 (green) binding to mitotic chromosomes from S2 cells. Analysis as described above. RAD21 accumulates on mitotic chromosome arms in ∼80% of cells depleted for NAP1. (**C**) Binding of SA to mitotic chromosomes from wild type or *nap1^KO1^* larval brain cells. (**D**) Binding of RAD21 to mitotic chromosomes from larval brain cells. ∼85% of *nap1^KO1^* larval brain cells show accumulation of SA and RAD21 on mitotic chromosome arms.

Resolution of sister chromatids is initiated at prophase by the bulk removal of cohesin from mitotic chromosome arms [24]–[26]. Therefore, we examined chromosomal cohesin binding after knockdown of NAP1. We used antibodies against SA and RAD21 to visualize cohesin on mitotic chromosomes that were isolated from colchicine-treated S2 cells. Depletion of NAP1 caused a striking accumulation of cohesin on the mitotic chromosome arms (Figure 2A–B). In mock-treated cells, we could only detect cohesin binding to the centromeric regions of mitotic chromosomes. Likewise, chromosomes from homozygous *nap1^KO1^* larval brain cells were densely coated with cohesin, whereas on wild type chromosomes cohesin binding was limited to the centromers (Figure 2C–D). Thus, NAP1 is required for cohesin release during mitosis.

Interestingly, NAP1's sub-cellular localization is dynamic and changes during the cell cycle (Figure S2). During interphase NAP1 is distributed about equally between cytoplasm and nucleus in *Drosophila* embryos, but at prophase there is a strong increase in nuclear NAP1. By metaphase, NAP1 has dissociated from chromosomes, along with most SA, but by anaphase both re-associate. Thus, nuclear accumulation of NAP1 at prophase agrees well with its function in promoting cohesin release in early mitosis. Together, these results suggest that NAP1 promotes sister chromatid resolution by mediating cohesin release from the chromosome arms.

### NAP1 and cohesin share genomic loci

**Figure 3:**
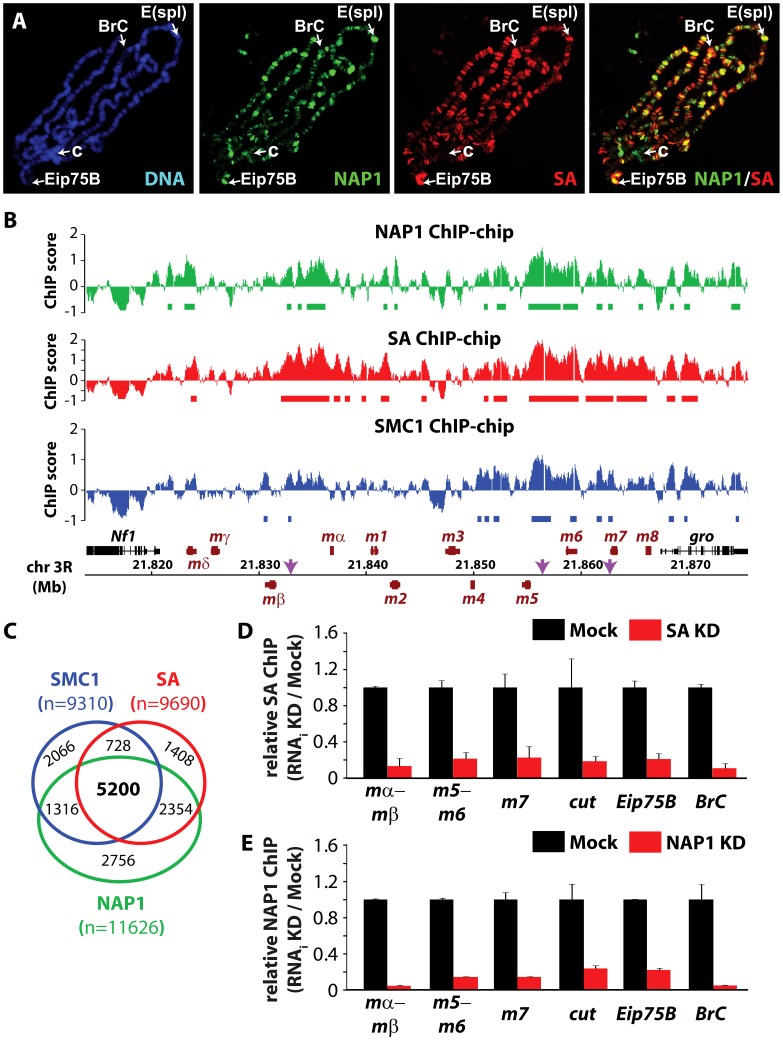
NAP1 and cohesin co-localize on chromatin. (**A**) Distribution of NAP1 and SA proteins on *Drosophila* salivary gland polytene chromosomes visualized by indirect immunofluorescence with antibodies against NAP1 (green) and SA (red). DNA was stained with 4′,6-diamidino-2-phenylindole (DAPI; blue). Split images and merge for red and green channels are shown. Regions harboring the *Enhancer of split* (*E(spl)*) gene cluster, *Broad Complex* (*BrC*), *Eip75B* and chromocenter (c) are indicated. (**B**) Genomic view of NAP1 (green), SA (red) and SMC1 (blue) ChIP-chip enrichment profiles at *Enhancer of Split E(spl)* NOTCH inducible gene cluster. Filtered binding sites (FDR<0.01) are indicated as bars below the respective profiles. ChIP-chip enrichment scores, genomic coordinates and genes are indicated. Regions examined by ChIP-qPCR are indicated by arrows. (**C**) Venn diagram depicting the overlap between SMC1, SA and NAP1 binding loci. The overlap between SMC1 and SA loci is 3.7 times greater (p<0.001) than expected by random chance. The overlap between NAP1 and cohesin loci derived from shared SMC1 and SA filtered peaks is 5.2 times greater (p<0.001) than expected by random chance. (**D**) ChIP-qPCR analysis of SA binding to genomic sites harboring *E(spl)*, *cut*, *Eip75B* and *BrC* genes selected from ChIP-chip profiles (Figure 3B and Figure S3A–C). ChIP enrichments after SA (red bars) and Mock (black bars) RNAi knockdowns (KD) were expressed relative to signals from mock-treated cells. For mock treatment we used dsRNAs directed against GFP. Results are based on 3 biological replicates and error bars represent standard error of mean (S.E.M.). (**E**) ChIP-qPCR analysis of NAP1 binding to genomic loci. Analysis as described above.

We wondered if NAP1 interacts with- and co-localizes with cohesin on chromatin. Immunostaining of interphase 3^rd^ instar larval salivary gland polytene chromosomes with antibodies against NAP1 and SA revealed a substantial overlap in their genomic binding loci (Figure 3A). For a high resolution analysis, we performed chromatin immunoprecipitations (ChIPs) in asynchronously dividing S2 cells using antibodies against NAP1, SA and SMC1. Following ChIP, isolated DNA fragments were mapped back to the genome by hybridization to *Drosophila* tiling arrays (Figure 3B and Figure S3A–C). All ChIPs were performed using 2 independent biological replicates, which showed a high degree of correlation (Figure S3D). The averaged genomic binding profiles of NAP1 and cohesin were highly correlated (r>0.7), indicating significant co-occupancy (Figure S3E).

We selected ChIP-chip peaks at a false discovery rate (FDR)<0.01, based on random permutation, yielding roughly 10,000 assigned binding loci for each factor. Intersection of genomic binding sites revealed a substantial overlap between cohesin and NAP1 loci (Figure 3C). Shared target loci include the NOTCH-regulated *cut* and *E(spl)* genes, and the ecdysone-controlled *BrC* and *Eip75B* genes (Figure 3B and Figure S3A–C). Binding of NAP1 and SA to these loci was confirmed independently by ChIP followed by quantitative PCR (Figure 3D–E). Previously, it was found that NAP1 and cohesin are required for the repression of NOTCH-target genes [15], [49], indicating that these factors might also cooperate in gene regulation.

### NAP1 interacts with cohesin

**Figure 4:**
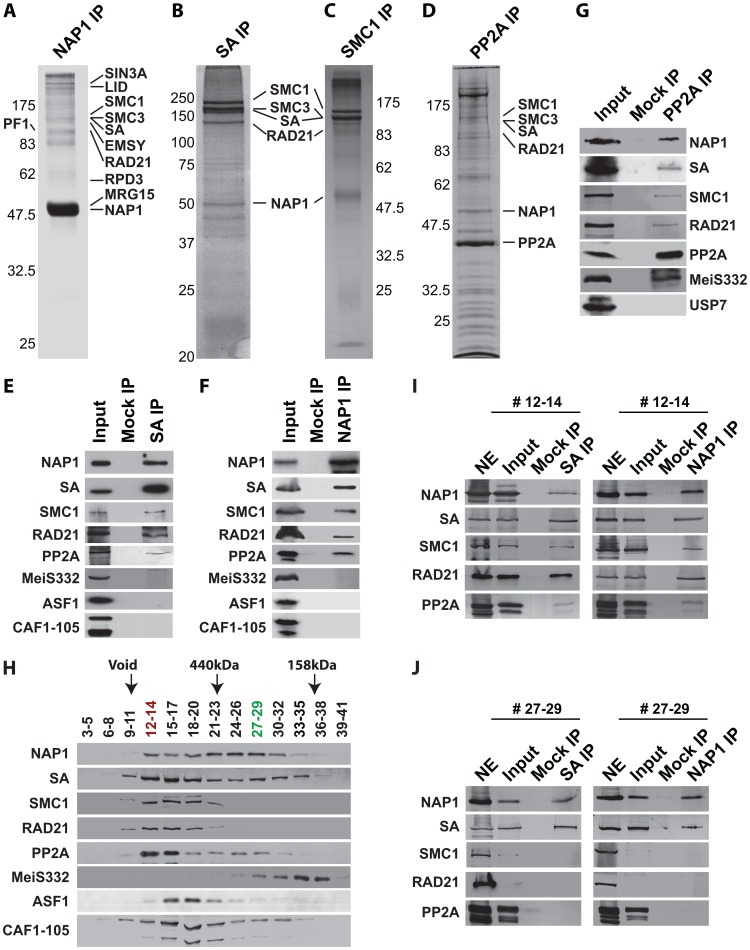
NAP1 interacts biochemically with the core cohesin complex and PP2A. (**A**) Proteomic analysis of the NAP1 interaction network. NAP1 and associated proteins were immunopurified from 0–12 hour *Drosophila* embryo nuclear extracts (NE) using affinity-purified antibodies raised against NAP1. After extensive washes with a buffer containing 600 mM KCl and 0.1% NP40, bound proteins were resolved by SDS-PAGE, visualized by coomassie staining and identified by mass-spectrometry. NAP1, RLAF subunits (SIN3A, LID, EMSY, PF1, RPD3 and MRG15) and cohesin subunits (SMC1/3, SA and RAD21) are indicated. A comprehensive list of identified proteins is provided in Table S1. (**B**) Identification of SA-associated factors. For a complete list of associated factors see Table S1. (**C**) Identification of SMC1-associated factors (see Table S1). (**D**) Identification of PP2A interaction network. Purification of PP2A-associated factors was performed as described above with antibodies against the catalytic subunit. For a list of selected factors associated with PP2A see Table S2. Protein bands corresponding to PP2A catalytic subunit, NAP1 and cohesin subunits, identified by mass spectrometric analysis are indicted. (**E**) SA was IPed from NE, followed by extensive washes with a buffer containing 600 mM KCl and 0.1% NP40. The binding of NAP1, cohesin subunits, PP2A, MeiS332, and histone chaperones ASF1 and CAF1 was assayed by immunoblotting using the appropriate antibodies. Mock IPs were performed with pre-immune serum. Input represents 10% of the binding reactions. A lack of SA association with MeiS332 was also confirmed under lower stringency (200 mM KCl, 0.1% NP40). (**F**) Co-IP analysis of NAP1 interactions. Analysis as described above. (**G**) Co-IP analysis of PP2A interactions. All PP2A interactions were detected under high stringency (600 mM KCl), except for MeiS332, which can be detected only at lower stringency (200 mM KCl). (**H**) Sephacryl S-300 size-exclusion chromatography analysis of NAP1, cohesin, PP2A and MeiS332. The indicated fractions were resolved by SDS-PAGE followed by immunoblotting. NAP1, PP2A and cohesin subunits SA, SMC1 and RAD21 co-eluted in column fractions corresponding to an apparent molecular mass of ∼1.5 MDa. In addition, NAP1 and SA, but not SMC1 or RAD21, eluted in lower molecular weight fractions of ∼300 kDa. Voided volume (void), determined by Blue Dextran 2000, and elution of the markers ferritin (440 kDa) and aldolase (158 kDa) are indicated. (**I**) co-IPs of NAP1 and SA from pooled high molecular weight S-300 column fractions (#12–14). IPs were performed as above. Input represents 10% of the binding reactions. (**J**) co-IPs of NAP1 and SA from pooled lower molecular weight fractions (#27–29). As S-300 fractions #27–29 lack SMC1 and RAD21, NE was used as a control for Western blotting efficiency.

To establish whether NAP1 interacts with cohesin, we immunopurified NAP1, SA and SMC1 from *Drosophila* embryo nuclear extracts (NE). Following extensive washes with a buffer containing 600 mM KCl and 0.1% NP40, immuno-purified proteins were resolved by sodium dodecyl sulfate polyacrylamide gel electrophoresis (SDS-PAGE) and visualized by Coomassie staining (Figure 4A–C). Protein identities were determined by mass spectrometry. Table S1 provides an overview of the proteins identified. In addition, we included the results of NAP1 purified from embryo NE using an independent antibody and NAP1 purified from S2 cells. All three independent NAP isolations yielded similar results. NAP1-associated proteins, such as the RLAF silencing complex [15], are mostly involved in transcription control. Importantly, the full cohesin complex was associated with NAP1 in all three independent purifications. Conversely, NAP1, but not RLAF, was identified alongside the cohesin subunits in both SA and SMC1 purifications. The majority of cohesin-bound proteins we identified have been implicated in cohesin biology, including the loading factors Nipped-B/SCC2 and MAU2/SCC4 [50]–[52]. Both NAP1 and cohesin purifications contained replication factor C, which is involved in DNA replication and cohesin loading [53]–[54]. We also noted the presence of protein phosphatase PP2A in the NAP1, SA and SMC1 purifications (Table S1). Immunopurification of PP2A from embryo NE followed by mass spectrometry revealed that PP2A is part of an extensive network of kinases and phosphatases (Figure 4D and Table S2). Most relevant for the present study, NAP1, the full cohesin complex, and cohesin loading factors Nipped B and Mau2 were all present in the PP2A purification.

We confirmed the specific association between NAP1, cohesin and PP2A by a series of co-immunoprecipitations (co-IPs) followed by immunoblotting. NAP1 was prominent in SA IPs, together with other cohesin subunits (Figure 4E). Conversely, the cohesin subunits SA, SMC1 and RAD21 were readily detected in NAP1 IPs (Figure 4F). Likewise, NAP1 and cohesin subunits were also detected in PP2A IPs under stringent conditions (600 mM KCl, 0.1% NP40). In addition, PP2A co-IPs under milder conditions (200 mM KCl, 0.1% NP40) with MeiS332, a *Drosophila* homolog of Sgo. Thus, the association between PP2A and Sgo/MeiS332 seems to be conserved from mammals to flies (Figure 4G). Other histone chaperones such as CAF1 or ASF1 did not bind cohesin, supporting the selectivity of NAP1 binding.

Protein-protein interaction assays using recombinant proteins confirmed the ability of NAP1 to bind cohesin subunits (Figure S4). NAP1 did not bind to the recombinant catalytic or regulatory subunits of PP2A, suggesting that they do not interact directly (Figure S4). In cells, the association between PP2A and NAP1 might be mediated through bridging by cohesin. Collectively, these results established a biochemical interaction between the histone chaperone NAP1 and cohesin.

### NAP1, but not PP2A, forms a separate module with SA

To characterize the NAP1, cohesin and PP2A interaction further, we used Sephacryl S-300 size-exclusion chromatography (Figure 4H). NAP1 and cohesin subunits co-eluted in column fractions corresponding to an apparent molecular mass of ∼1.5 MDa. A substantial portion of NAP1 and SA, but not the other cohesin subunits, were also present in fractions corresponding to an average molecular mass of ∼300 kDa. We did not detect appreciable amounts of NAP1 or SA eluting at their predicted molecular weights, suggesting they are not present as free proteins. Co-IPs from pooled high molecular weight S-300 column fractions 12–14 confirmed the presence of a large assemblage harboring the full cohesin complex, NAP1 and PP2A (Figure 4I). In addition, co-IPs from the pooled lower molecular weight fractions 27–29 revealed a separate complex comprising NAP1 and SA, but devoid of PP2A or the other cohesin subunits (Figure 4J). A possible interpretation of these results is that NAP1 forms a separate module with SA, which interacts dynamically with the full cohesin complex.

### NAP1 counteracts PP2A association with cohesin

**Figure 5:**
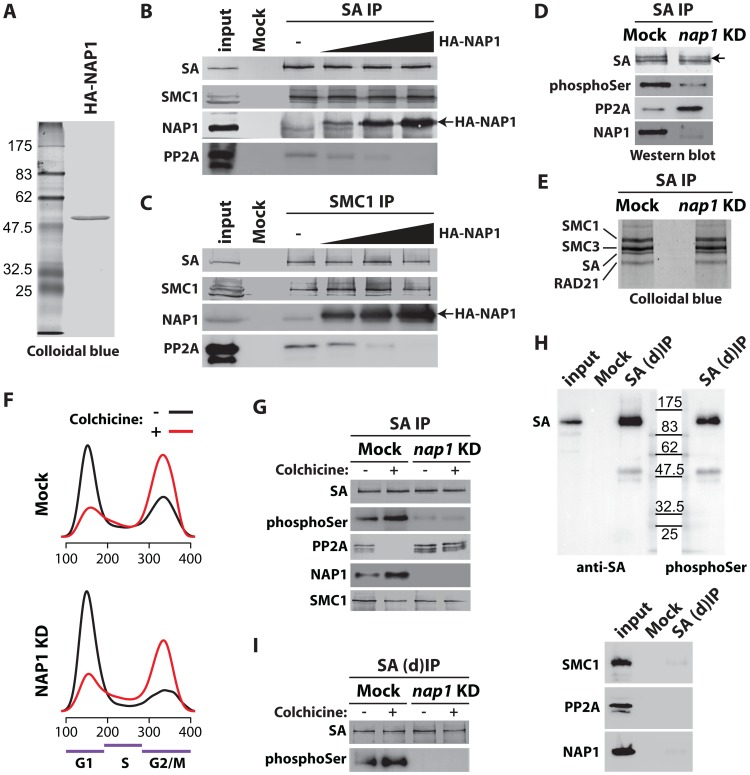
NAP1 regulates SA phoshorylation levels by counteracting PP2A association with chromosomal cohesin during mitosis. (**A**) Colloidal blue staining of immunopurified, baculovirus expressed HA-tagged NAP1 from Sf9 cells. (**B–C**) NAP1 can displace PP2A from cohesin. The endogenous cohesin complex was immunopurified from embryo NE with antibodies against SA (**B**) or SMC1 (**C**) as described in Figure 4B–C. Next, increasing amounts of purified HA-NAP1 was added. Following extensive washes the binding of endogenous NAP1, HA-NAP1 and PP2A to the cohesin complex was analyzed by immunoblotting. (**D**) Western blot analysis of SA IPed from either mock-treated or NAP1 knockdown (KD) cells. Blots were probed with antibodies against SA, phosphorylated serine (phosphoSer), PP2A or NAP1. Note the increased PP2A binding to SA in the absence of NAP1. Concomitantly, SA phosphorylation levels decreased, as revealed by the antibodies against phosphoSer, which recognize a band corresponding to the migration of SA. A slower migrating form of SA, presumably due to phosphorylation, is indicated by an arrow. (**E**) NAP1 depletion does not affect cohesin complex stability or stoichiometry. In parallel to the immunoblotting in (**D**), we resolved the IPed SA by SDS-PAGE followed by colloidal blue staining. The identity of the cohesin subunits were determined by mass spectrometric analysis (Figure S5A). (**F**) Cell cycle profiles of mock-treated (Mock) or NAP1 depleted (KD) S2 cells arrested in mitosis by colhicine (red curves) as compared to asynchronously dividing cells (black curves). Cell cycle profiles were determined by FACS analysis. G1, S and G2/M phases are indicated. (**G**) PP2A dissociates from cohesin in mitosis, whereas NAP1 binding to SA is increased. Immunoblotting analysis of SA IPed from either mock or NAP1 depleted (KD) cells, treated (+) or untreated (−) with colhicine as in (**D**). Similar results were obtained for SMC1 IPs from colhicine-treated cells (Figure S6). (**H**) Immunopurification of SA from S2 cell extracts denatured by 6M Urea ((d)IP) to selectively identify phosphorylated SA with antibodies against phosphorylated serine (phosphoSer). Note that SMC1, NAP1 and PP2A dissociate from SA under these conditions. (**I**) Western blot analysis of SA IPed under denaturing conditions ((d)IP) from either mock- or NAP1 depleted (KD) cells, which were either treated (+) or untreated (−) with colchicine, confirmed the changes in SA phosphorylation caused by mitotic arrest or NAP1 depletion.

The removal of cohesin from chromosome arms is triggered by SA phosphorylation in prophase, whereas centromeric cohesin is protected from phosphorylation by a complex of PP2A and Sgo/MeiS332 [32]–[34], [39]–[40]. The results from our interaction studies (Figure 4) suggest that NAP1, or the NAP1-SA module, may compete with PP2A for cohesin binding. Therefore, we considered the possibility that NAP1 promotes cohesin dissociation from chromosome arms by blocking PP2A binding to cohesin. To test this idea, we performed competition assays using recombinant NAP1 expressed in baculovirus (Figure 5A) and cohesin complex immunopurified from embryo nuclear extracts with antibodies against SA or SMC1 (Figure 4B–C). Recombinant NAP1 binds immunopurified cohesin and drives the dissociation of endogenously bound PP2A (Figure 5B–C). To test the effect of NAP1 on PP2A binding to cohesin *in vivo*, we immunopurified cohesin complex from S2 cells that were either mock treated or depleted for NAP1. Loss of NAP1 caused a strong increase in PP2A association with cohesin (Figure 5D). Accompanying the increase in cohesin-bound PP2A, immunoblotting with antibodies directed against phospho-Serine or SA revealed decreased levels of phosphorylated SA following NAP1 knockdown. Depletion of NAP1 affected neither the integrity, nor the stoichiometry of the cohesin complex (Figure 5E and Figure S5A). Likewise, cellular levels of PP2A were not affected by NAP1 depletion (Figure S5B).

Next, we investigated if NAP1 counteracts PP2A binding to cohesin during mitosis. Immunopurification of SA and SMC1 revealed increased association of NAP1 with cohesin in colchicine treated cells that are arrested in mitosis, compared to untreated cells (Figure 5F–G and Figure S6). Whereas NAP1 associates, PP2A dissociates from cohesin in mitosis allowing persistence of the phosphorylation of SA by mitotic kinases. After NAP1 knockdown, PP2A stayed bound to cohesin in mitotic cells and SA remained dephosphorylated suggesting that NAP1 drives PP2A dissociation (Figure 5G and Figure S6). To ensure the phosphorylation effects we observe are SA specific, we immunopurified SA from S2 cells under denaturing conditions. Immunoblotting with antibodies against phospho-Serine confirmed that we detect phosphorylated SA and not an associated factor such as SMC1, which dissociates from SA under these conditions (Figure 5H). Finally, the effect of colchicine treatment or NAP1 knockdown show that SA phosphorylation is cell-cycle regulated and depends on NAP1 (Figure 5I). We conclude that NAP1 counteracts PP2A binding to cohesin, thereby preventing SA dephosphorylation during G2/M transition, resulting in a net increase in phosphorylated SA.

### NAP1 counteracts PP2A binding to chromosomes

**Figure 6:**
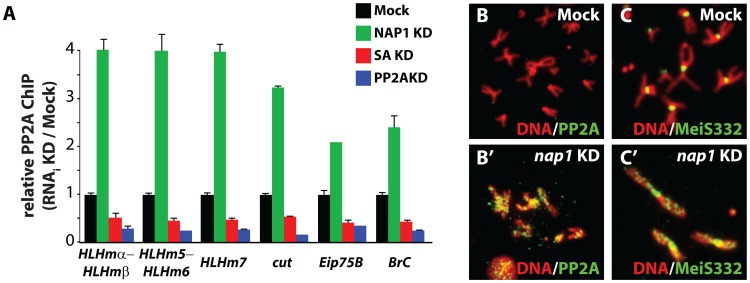
NAP1 counteracts PP2A association with chromatin. (**A**) ChIP-qPCR analysis of PP2A binding to the genomic loci of NAP1 and cohesin. PP2A was ChIPed from mock-treated S2 cells, or cells depleted for either NAP1 (green), SA (red) or PP2A itself (blue). ChIP signals for all knockdowns (including Mock KD) were expressed relative to the mock-treated S2 cells. ChIPs were performed using 3 biological replicates and error bars represent S.E.M. (**B**) Loss of NAP1 leads to PP2A accumulation on the arms of mitotic chromosomes. Indirect immunofluorescent analysis of PP2A (green) binding to mitotic chromosomes of mock-treated S2 cells (**B**) or after NAP1 knockdown (**B′**). DNA visualized by DAPI staining is shown in red. (**C**) Accumulation of MeiS332 on the arms of mitotic chromosomes after NAP1 knockdown. MeiS332 localizes at the centromeres of mitotic chromosomes of mock-treated S2 cells (**C**), but spread onto the chromosome arms after loss of NAP1 (**C′**).

To examine the role of NAP1 in the binding of PP2A to chromosomal cohesin loci, we performed ChIPs (Figure 6A). Knockdown of NAP1 resulted in a striking increase in PP2A association with the cohesin and NAP1 binding sites examined. In contrast, SA depletion caused a loss of PP2A binding to the genomic NAP1 and cohesin sites, suggesting that SA tethers PP2A to chromatin. Confirming the specificity of the assay, ChIP signals were strongly reduced after PP2A knockdown. Based on these ChIP results, we conclude that PP2A binding to chromosomal cohesin is attenuated by NAP1. This notion is supported by immunostaining of mitotic chromosomes. Knockdown of NAP1 caused a strong accumulation of PP2A onto the chromosome arms in ∼80% of cells (Figure 6B). Upon loss of NAP1, MeiS332 is no longer restricted to the centromers, but now coats the chromosome arms (Figure 6C). Thus, MeiS332 behaves similar to its centromeric partner PP2A. This result provides additional support for the notion that the role of PP2A-Sgo/MeiS332 in mitosis is conserved from flies to mammals. Collectively, our findings suggest that NAP1 regulates sister chromatid resolution by preventing PP2A binding to cohesin on chromosome arms. Blockage of PP2A allows phosphorylation of SA by mitotic kinases, which drives cohesin release and sister chromatid resolution.

### NAP1 and PP2A are antagonistic regulators of sister chromatid resolution

**Figure 7:**
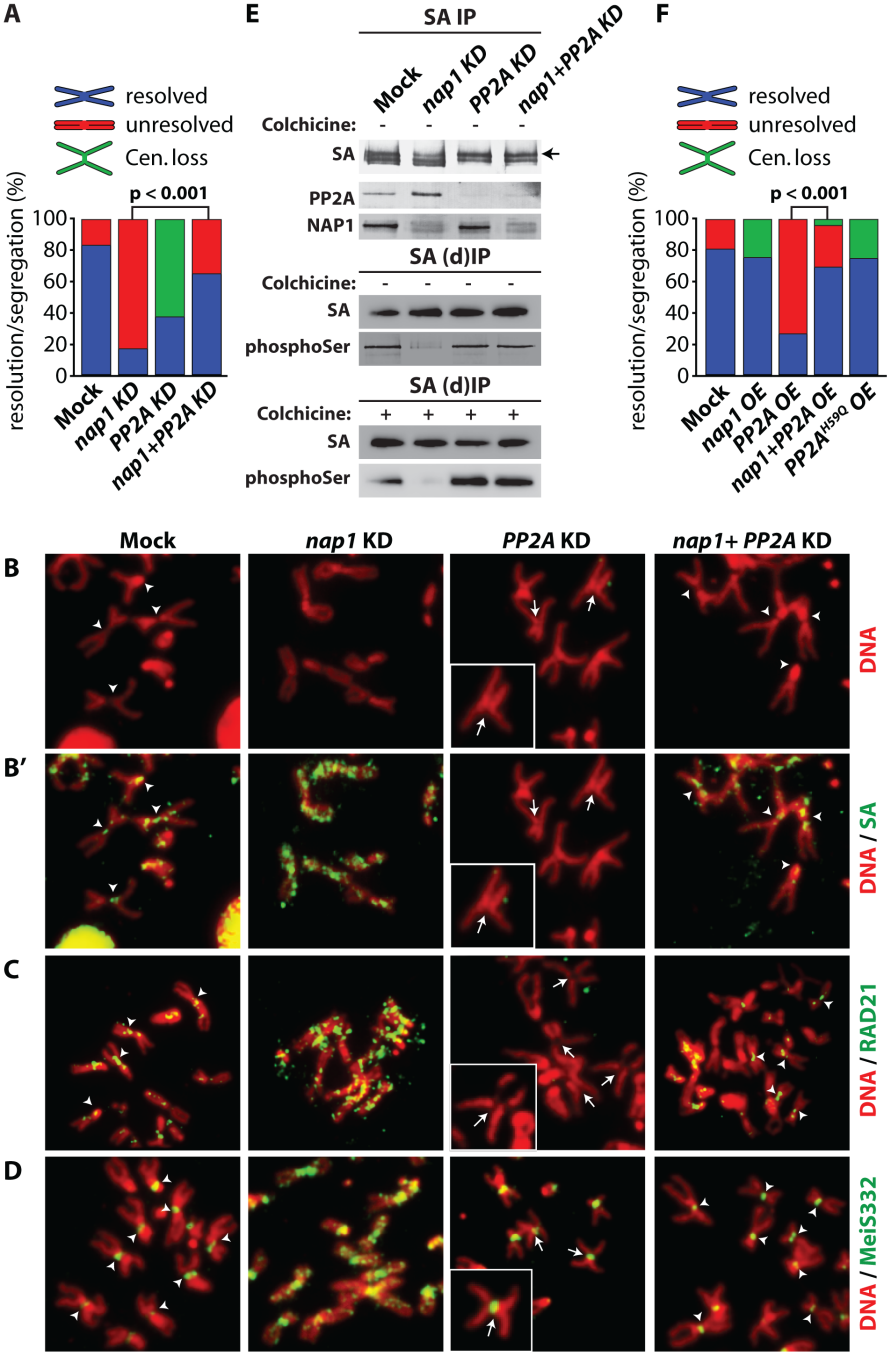
NAP1 and PP2A act antagonistically in cohesin cycle. (**A**) Analysis of mitotic chromosomes from colchicine-treated S2 cells after knockdown of NAP1, PP2A or both factors. We quantified the frequency of resolved (blue), unresolved (red) sister chromatids and loss of centromeric cohesion (Cen. Loss; green). Concomitant depletion of NAP1 and PP2A resulted in a statistically significant increase of the frequency of resolved chromatids compared to the NAP1 knockdown, as determined by χ^2^-test (n>30, from 3 biological replicates). For the corresponding Western blot analysis see Figure S7A. (**B**) Representative example of mitotic chromosomes from colhicine-treated S2 cells depleted for NAP1, PP2A or for both proteins. DNA visualized by DAPI staining is shown in red. Centromers are indicated by arrowheads, whereas loss of centromeric cohesion is indicated by full arrows. (**B′**) The localization of SA (green) on mitotic chromosomes same as in (**B**) was determined by indirect immunofluorescence. (**C**) RAD21 (green) localization on mitotic chromosomes. (**D**) MeiS332 (green) localization on mitotic chromosomes. (**E**) Depletion of PP2A restores SA phosphorylation in cells lacking NAP1. Western blot analysis of SA IPed from either mock-treated S2 cells or after knockdown (KD) of NAP1, PP2A or both proteins under normal (top panel) or denaturing (middle panel, (d)IP) conditions from asynchronously dividing cells (− colhicine) or colhicine treated cells (bottom panel, + colhicine). Blots were probed with antibodies against SA, phosphorylated serine, PP2A or NAP1. After NAP1 knockdown, SA phosphorylation levels drop substantially. Whereas depletion of PP2A alone does not affect bulk SA phosphorylation, concomitant knockdown of PP2A and NAP1 neutralized the effect of NAP1 depletion, leading to restored levels of phosphorylated SA. Antibodies against phosphoSer recognize a band corresponding to the migration of SA. A slower migrating form of SA, presumably due to phosphorylation, is indicated by an arrow. (**F**) Analysis of mitotic chromosomes from colchicine-treated S2 cells after over-expression (OE) of GFP (Mock), NAP1, PP2A, both NAP1 and PP2A or the catalytic mutant PP2A^H59Q^. Quantification of mitotic phenotypes was as described above (A). Overexpression of PP2A, but not PP2A^H59Q^, resulted in significant increase of the frequency of unresolved chromatids. The PP2A over-expression phenotype was rescued by co-expression of NAP1, as determined by χ^2^-test (n>30, from 3 biological replicates). For the corresponding Western blot analysis see . Representative examples of mitotic chromosomes are shown in Figure S7C–D.

If NAP1 mediates sister chromatid resolution by counteracting PP2A, concomitant loss of PP2A should reverse the effects of NAP1 depletion. To test this idea, we analyzed mitotic chromosomes after knockdown of either NAP1, PP2A or both factors (Figure 7A and Figure S7A). First, we observed that the effect of PP2A knockdown is the opposite of NAP1 depletion. Instead of unresolved sister chromatids, which are the hallmarks of NAP1 depletion, knockdown of PP2A caused diminished centromeric cohesion in ∼65% of mitotic cells (Figure 7A–B). This was accompanied by dissociation of centromeric SA and RAD21, but not MeiS332 in ∼60–65% of mitotic cells (Figure 7B–D). However, after concomitant depletion of NAP1 and PP2A the majority of mitotic chromosomes appeared normal. We observed no loss of centromeric cohesion and the majority of sister chromatids were resolved properly. In agreement with the rescued phenotype, cohesin and MeiS322 localization was largely normal in ∼70–80% of cells depleted for both NAP1 and PP2A.

Our earlier biochemical results indicated that NAP1 counteracts PP2A binding to SA, thereby preventing SA dephosphorylation. Therefore, we tested if the loss of phosphorylated SA after NAP1 knockdown was dependent on PP2A. Indeed, concomitant knockdown of PP2A and NAP1 restored the levels of phosphorylated SA, Whereas depletion of PP2A alone did not affect SA phosphorylation (Figure 7E). These results suggest that NAP1 and PP2A act antagonistically in the control of the cohesion cycle.

To complement the experiments in which we depleted endogenous proteins, we compared the effects of ectopic expression of NAP1, PP2A or the PP2A^H59Q^ catalytic mutant [55] in S2 cells (Figure 7F and Figure S7B–D). Over-expression of NAP1 resulted in a mild increase of defective centromeric cohesion and a loss of cohesin binding to the centromers. Ectopic expression of PP2A gave the opposite phenotype, and mimicked the effect of NAP1 depletion, namely, cohesive linkage of the majority of mitotic chromosome arms (∼80%). Highlighting the importance of PP2A's catalytic activity, expression of PP2A^H59Q^ did not lead to failed sister chromatid resolution, but a mild loss of centromeric cohesion. Thus, ectopic expression of a phosphatase-defective PP2A mutant yielded a similar phenotype as depletion of endogenous PP2A. When over-expressed together, NAP1 and PP2A cancelled each other out, resulting in wild type mitosis in most cells. The results of these ectopic expression assays are fully consistent with those of our depletion experiments. Collectively, they demonstrate the antagonistic function of NAP1 and PP2A in regulation of SA phosphorylation, cohesin release and sister chromatid resolution.

## Discussion

As reflected by their name, a major activity of histone chaperones is to prevent illicit liaisons and guide newly synthesized histones to sites of chromatin assembly. Here, we described a mitotic function for the canonical histone chaperone NAP1 that is unrelated to nucleosome assembly. We found that NAP1 binds cohesin and blocks dephosphorylation of SA by PP2A, thereby promoting cohesin dissociation from the chromosome arms. Consequently, chromosomal binding of cohesin during mitosis is controlled by the balance between the opposing activities of NAP1 and PP2A.

NAP1 is part of a large assemblage including the full cohesin complex and PP2A. In addition, NAP1 and SA form a subcomplex, which lacks the other cohesin subunits and PP2A. An attractive scenario is that the NAP1-SA module or NAP1 alone competes with PP2A-bound SA within the full cohesion complex. PP2A displacement by NAP1 allows stable phosphorylation of cohesin and its dissociation during early mitosis. NAP1 might also act as a direct inhibitor of PP2A catalytic activity, because a mammalian NAP1 homolog, SET, has been identified as a potent PP2A inhibitor [56], which promotes sister chromatid segregation during mouse oocyte miosis [57]–[58]. In addition, NAP1 might help cohesin phosphorylation by tethering Polo kinase to cohesin. In fact, we detected a potential association between NAP1 and Polo kinase (Figure S4). However, the dramatic chromosome condensation defects after Polo kinase depletion precluded a functional evaluation of a possible role of NAP1 in its function. Nevertheless, although we cannot exclude additional NAP1 activities, our functional experiments established that blockage of PP2A suffices to explain the crucial role of NAP1 during sister chromatid resolution.

NAP1 not only regulates the chromosomal distribution of cohesin and PP2A, but also that of MeiS332, a fly homolog of Sgo. The function of MeiS332 and PP2A appears to be largely conserved from mammals to flies because they bind each other and depletion of either factor causes a loss of centromeric cohesion [39]–[45]. Either knockdown of NAP1 or over-expression of PP2A (Figure S7E) caused spreading of MeiS332 onto the arms of mitotic chromosomes, accompanying the loss of sister chromatid resolution. Thus, the balanced antagonism between NAP1 and PP2A controls chromosomal association of both cohesin and MeiS332 during mitosis.

One level of regulation involves changes in NAP1's subcellular localization and chromatin binding through the cell cycle. At prophase there is a strong increase in the level of nuclear NAP1, but by metaphase, NAP1 and cohesin have dissociated from the chromosomes (Figure S2). Thus, the dynamic behavior of NAP1 correlates well with its function in promoting cohesin release at early mitosis. Regulation of NAP1 localization may involve cyclin B-cdc2/cdk1 kinase complexes. Previously it was found that yeast and vertebrate NAP1 are phosphorylated by cyclin B-cdc2 [59] and that yeast cyclin B requires NAP1 for its full range of mitotic functions [59].

We suggest that histone chaperones are at the hubs of specialized protein networks that perform a wide variety of tasks in chromosome biology. Through association with distinct partners, NAP1 is able to perform different functions. By acting as a histone chaperone, NAP1 mediates chromatin assembly [60]–[61]. Through recruitment of the histone H3 deacetylase and H3K4 demethylase complex RLAF, NAP1 controls gene-selective silencing at developmental loci [15]. Finally, by binding cohesin and blocking SA dephosphorylation by PP2A, NAP1 mediates sister chromatid resolution during mitosis. These results emphasize the surprisingly diverse- and specific regulatory functions of histone chaperones in chromosome biology.

## Materials and Methods

### Antibodies

Anti-SA antibodies were raised in guinea pigs and rabbits against a GST-fusion protein encoding SA amino acids 12–312, purified from E. Coli. Rabbit polyclonal antibodies against the NAP1 were previously described [15], anti-SMC1 and anti-RAD21 antibodies were a gift from D. Dorsett [49], anti-phospho-Serine (Abcam, ab6639), anti PP2A-C (BD Biosciences, 610555), anti-CID (Abcam, ab10887) and anti-α-Tubulin (Sigma, T5168).

### S2 cell culture, RNAi knockdown and cell transfection

*Drosophila* S2 cells were cultured in Schneider's media (Invitrogen 21720-024) supplied with 10% FBS. Double-stranded RNAs for NAP1, SA and PP2A/MTS were synthesized using an Ambion Megascript T7 kit according to the manufacturer's protocol with the following primers: 5′-TATTGAACAATGGACGCCC-3′ and 5′-TGAAACTCCAAGGTGTACG-3′ for NAP1; 5′- CAGTCAAATACATAAAATGATGGCG-3′ and 5′-GCTCAATCCATTGGTCAACA-3′ for SA; and 5′-GGCAGTCTTTCCCTTCGTATATC-3′ and 5′-CGAACTTGTGTCTCTGTCAACTG-3′ for the PP2A catalytic subunit encoded by the *mts* gene. Primers were flanked by T7-promoter sequence (5′-TTAATACGACTCACTATAGGGAGA-3′) at the 5′-end. For mock knockdowns, dsRNA against GFP was synthesized with the following primers (5′- CAAGAGTGCCATGCCCGAAGGT-3′ and 5′-TGTGGTCACGCTTTTCGTTGGG-3′) flanked by the T7 promoter sequence. For cytological or FACS analysis, S2 cells were incubated with dsRNA for 2 days as described [62]. Open reading frames (ORFs) of GFP, NAP1, PP2A and catalytically inactive PP2A - PP2A^H59Q^ [55] were cloned into pENTR/DTOPO entry vector (K2400-20, Invitrogen) and subcloned into *Drosophila* expression vector pAHW (obtained from the Drosophila Genome Resource Center, DGRC) carrying the Actin 5C promoter and a sequence encoding the HA-tag by LR-clonase reaction. Polyethylenimine (PEI) ∼25000 Da (408727 Sigma-Aldrich) was used for transient transfection of S2 cells as described [63]. Protein expression and mitotic chromosomes were analyzed 48 hours post-transfection.

### Cytological procedures

For indirect immunofluorescence analysis, S2 cells were fixed in phosphate buffered saline (PBS) buffer containing 3.7% formaldehyde for 5 min. Cells were incubated with antibodies against CID and α-Tubulin diluted at 1∶200 and 1∶1000, respectively. After washes with PBS, cells were incubated with fluorescently-labeled secondary antibodies (Molecular Probes) and analyzed with the Leica FW4000 imaging system. For mitotic chromosomes preparation from S2 cells and *Drosophila* larvae brain cells, cells were treated with 10 mM colhicine for 10 min, incubated with 0.5% sodium citrate for 5 min (10 min for brain cells) and fixed in 45% acetic acid containing 3.7% formaldehyde for 5 min. Next, cells were squashed on objective slides and covered by a cover-slip. Slides were washed with PBS, and incubated with anti-SA, anti-RAD21 and anti-MeiS332 antibodies, each diluted 1∶200, and anti-PP2A diluted 1∶20. After washes and incubation with fluorescent-labeled secondary antibodies, mitotic chromosomes were analyzed on a Leica FW4000 imaging system. To quantify the accumulation or loss of cohesin, PP2A or MeiS332 on mitotic chromosomes, chromosomes from at least 30 cells were analyzed. Fluorescent assisted cell sorting (FACS) of S2 cells was performed as described [64].

### Chromatin immunoprecipitation (ChIP)

For chromatin immunoprecipitation experiments (ChIPs), S2 cells were fixed with 1% formaldehyde for 10 min. Fixation was stopped by addition of 125 mM glycine and cells were lysed in ice-cold L buffer (1% sodium dodecyl sulfate, 10 mM EDTA, 50 mM Tris-HCl pH 8.1, 0.5 mM phenylmethylsulfonyl fluoride - PMSF, and 100 ng/ml of leupeptin and aprotinin). Cross-linked chromatin was fragmented by sonication to an apparent length of ∼500 bp. Cross-linked chromatin (100 µg) was diluted with 9 volumes of buffer D (150 mM NaCl, 20 mM Tris-HCl pH 8.1, 2 mM EDTA pH 8.0, 1% Triton-X100, 0.5 mM PMSF, and 100 ng/ml of leupeptin and aprotinin) and pre-cleared with 10 µl of protein A agarose (16–157, Upstate). Pre-cleared chromatin was incubated at 4°C with appropriate antibodies overnight and precipitated with 20 µl of protein A agarose. For Mock ChIPs, chromatin was incubated with preimmune serum. Protein A agarose was washed extensively with buffer W (20 mM Tris-HCl pH 8.1, 2 mM EDTA pH 8.0, 0.1% SDS, 1% Triton X-100, 0.5 mM PMSF, and 100 ng/ml of leupeptin and aprotinin) containing 150 mM NaCl, and 1× with buffer W/500 mM NaCl. DNA retaining on the protein A agarose was eluted by incubating with 250 µl buffer E (1% SDS, 0.1 M NaHCO_3_, 500 µg/ml Proteinase K) for 2 hrs at 37°C and overnight at 65°C and extracted with QIAquick PCR purification kit (Qiagen Cat. 28106). DNA isolated from the ChIPs was analyzed by quantitative PCR (ChIP-qPCR) using the Bio-Rad CFX96 Real-Time System. Sequences of the primers used for qPCR are listed in Table S3.

For ChIP-chips, recovered DNA was amplified with REPLI-g (Qiagen Cat. 150025), digested with DNase, labeled and hybridized on Affymetrix *Drosophila* tiling 2.0R arrays. For each antibody we perform 2 independent biological replicates. ChIP-chip hybridization intensities were analyzed using R and R/Bioconductor packages as described [65]. In brief, log_2_(ChIP/Input) and log_2_(Mock/Input) ratios were quantile normalized in parallel. Then, averaged ratios were median scaled and log_2_(Mock/Input) ratio was subtracted from log_2_(ChIP/input) ratio resulting in ChIP-chip score. Finally, peaks were selected based on random permutations at false discovery rate (FDR)<0.01.

### Protein immunopurifications, mass spectrometry and co-immunoprecipitations

Nuclear extracts (NEs) from 0–12 hour old *Drosophila* embryos or S2 cells were prepared as described [66]. Immunopurification procedures were also performed essentially as described [66]. Briefly, for immunopurification of NAP1 and cohesin, extracts were incubated with affinity-purified antibodies cross-linked to protein A sepharose beads (GE Healthcare 17-0963-03) by dimethylpimelimidate. After incubation, beads were washed extensively with HEMG buffer: 25 mM HEPES-KOH pH 7.6, 0.1 mM EDTA, 12.5 mM MgCl_2_, 10% glycerol, a cocktail of protease inhibitors, and containing 600 mM KCl, 0.1% NP-40 (HEMG/600). Proteins retained on the beads were eluted with 100 mM NaCitrate buffer pH 2.5, resolved by SDS-PAGE and visualized by colloidal-blue staining or immunoblotting. For denaturing IPs of SA, cells were lysed in a buffer containing 6M Urea, 150 mM NaCl, 5 mM DTT and 50 mM Tris pH 7.6 followed by the step-wise dialysis to lower the Urea concentration to 2M. Finally, the extract were dialyzed against HEMG/400 and SA was immunoprecipitated as described above.

NE fractionation by (NH4)_2_SO4 precipitation, POROS-heparin and Sephacryl S-300 size-exclusion chromatography were performed as described [67]. In brief, NEs were concentrated by chromatography on a POROS-heparin (PerSeptive Biosystems) column equilibrated with HEMG/100 (pH 7.6) buffer followed by a step elution with HEMG/400 (pH 7.6) buffer (H0.4 fraction). The H0.4 fraction was loaded onto an 800-ml Sephacryl S-300 column (Pharmacia) equilibrated and developed with HEMG/100 (pH 7.6) buffer.

Mass spectrometry analysis of immunopurified protein complexes was performed on a LTQ-Orbitrap hybrid mass spectrometer (ThermoFischer) as described [68]. Detected peptides were matched against the FlyBase database (http://www.flybase.org/) using a Mascot search algorithm and identified proteins, Mascot scores and number of unique peptides are listed in Table S1 and Table S2. Details will be made available upon request.

### Recombinant NAP1 purification, interaction- and competition assays

Recombinant HA-tagged NAP1 (HA-NAP1) was expressed and purified using the baculovirus expression system. For competition assays, the cohesin complex bound to PP2A was immunopurified from embryo nuclear extracts with antibodies against SA or SMC1 as described above. Purified HA-NAP1 was incubated with the cohesin complex captured on Protein A beads followed by a series washes with HEMG/600. Proteins retained on the beads were resolved by SDS-PAGE and analyzed by immunoblotting. For interaction assays, HA-NAP1 captured on anti-HA coated beads was incubated with recombinant [^35^S]methionine-labeled cohesin subunits, PP2A subunits and Polo produced by in vitro transcription/translation system (Promega L1170). Proteins bound to NAP1 were resolved by SDS-PAGE and detected by autoradiography.

### Accession numbers

Data were deposited to Gene Expression Omnibus under accession number GSE30938 (http://www.ncbi.nlm.nih.gov/geo/query/acc.cgi?token=xnuhnwkmqqmwcli&acc=GSE30938).

## Supporting Information

Figure S1 NAP1 is required for cell proliferation and normal mitosis. (**A**) Left panel: immunoblotting analysis of S2 whole-cell extracts prepared from mock-treated cells (Mock) or cells depleted for either NAP1 or SA by RNAi-mediated gene knockdown (KD). Histone H3 served as a loading control. Note that there is a modest reduction in SMC1 and RAD21 protein levels in cells depleted for SA. Middle panel: analysis of NAP1 and cohesin protein levels in *Drosophila* larvae brain cells homozygous for the NAP1 knockout allele *nap1^KO1^* by immunoblotting with the indicated antibodies. Right panel: Western blot analysis of S2 whole-cell extracts prepared from mock-treated cells (Mock) or cells depleted for CAF1-105. (**B**) Proliferation of S2 cells treated with dsRNA directed against NAP1 is significantly reduced in comparison to the mock-treated cells. Cells were plated at 10^6^ cells/ml, incubated with dsRNA directed against GFP (Mock) or NAP1 (KD) and counted for 4 consecutive days. Error bars indicate standard error of mean obtained from 3 different experiments. (**C**) Cell cycle profiles of S2 cells after depletion of NAP1. S2 cells were treated with dsRNA directed against NAP1 (KD) or GFP (Mock) for 2 days. Cell cycle profiles were determined by fluorescent assisted cell sorting (FACS) analysis. Cells were fixed, and DNA was stained with propidium iodide (PI). Quantification is based on gated cells. The percentage of cells in G1, S or G2/M phases and corresponding FACS profiles are shown for mock-treated cells (black) and NAP1 knockdown cells (NAP1 KD, red). Depletion of NAP1 caused a mild accumulation of the cells in G1 phase, and an increase of cells with a DNA content >4n (marked by asterisk). (**D**) NAP1 knockdown causes mitotic defects. Indirect immunofluorescent analysis of *Drosophila* S2 cells with antibodies against tubulin (blue) and CID (green) was performed to visualize mitotic spindles and centromeres, respectively. DNA was stained by DAPI (red). Representative mitotic defects in NAP1 depleted cells, such as sister chromatid missegregation and misalignment relative to the equator, and DNA bridges during anaphase are indicated by arrowheads. The frequency of mitotic defects for NAP1 depleted (KD) cells is significantly higher than for mock-treated cells, as determined by a χ^2^-test of >30 cells analyzed for each knockdown (bottom panel). (**E**) NAP1 does not have an appreciable effect on histone levels detected on mitotic chromosomes. Indirect immunofluorescent analysis of histone H3 (green) on methanol-fixed mitotic chromosomes in mock-treated or NAP1 knockdown (KD) cells. DNA, visualized by DAPI, is shown in red.
(PDF)

Figure S2 Changes in NAP1's subcellular localization during mitosis. Indirect immunofluorescence analysis of *Drosophila* embryos stained with antibodies against NAP1 (red) and SA (green). DNA was visualized by DAPI (blue). SA is nucleus during interphase and pro(meta)phase. By metaphase, the bulk of SA is removed from the chromosome arms, but by anaphase it binds the chromosomes again. During interphase, NAP1 is distributed roughly equally between nucleus and cytoplasm. However, there is a strong increase in NAP1 nuclear localization at pro(meta)phase. During metaphase, NAP1 dissociates from the DNA, but still surrounds the mitotic chromosomes. During anaphase, NAP1 starts to re-associate with the chromosomes.
(PDF)

Figure S3 Genome-wide binding profiling of NAP1 and cohesin by ChIP-chip. (**A–C**) Genomic view of NAP1 (green), SA (red) and SMC1 (blue) ChIP-chip enrichment profiles across genomic regions harboring the *cut* NOTCH target gene (**A**), and two ecdysone-inducible loci harboring *Broad Complex* (*BrC*) (**B**) and *Eip75B* (**C**) genes. Filtered binding sites are indicated as bars below the respective profiles. ChIP-chip enrichment scores, genomic coordinates and genes are indicated. Regions examined by ChIP-qPCR are indicated by arrows. (**D**) The genome-wide ChIP-chip profiles of NAP1 and cohesin subunits SMC1 and SA are highly correlated between independent biological replicates. (**E**) The genome-wide ChIP-chip profiles of NAP1 and cohesin subunits SMC1 and SA are highly correlated to each other, in contrast to the binding profile of the ATP-dependent chromatin remodeler MI2. Pairwise smooth scatter plots of averaged NAP1, SA, SMC1 and MI2 ChIP-chip enrichment scores are shown, and correlations (r) and linear regression lines are indicated.
(PDF)

Figure S4 NAP1 interacts *in vitro* with cohesin subunits, but not PP2A. Protein-protein interaction assay using recombinant HA-tagged NAP1, expressed in Sf9 cells using the baculovirus system (Figure 5A). Cohesin subunits (SA, SMC1, SMC3 and RAD21), PP2A catalytic subunit (PP2A) and regulatory subunits (PP2A-29B, WDB), and Polo like kinase were produced using a coupled in vitro transcription/translation (IVT) system in the presence of [^35^S]methionine. Radiolabeled proteins were incubated with Protein A beads decorated with anti-HA antibodies bound to HA-NAP1 or lacking HA-NAP1 (Mock). Following extensive washes with a buffer containing 600 mM KCl and 0.1% NP40, proteins were resolved by sodium dodecyl sulfate polyacrylamide gel electrophoresis (SDS-PAGE) and detected by autoradiography. Input corresponds to 10% of the binding reaction.
(PDF)

Figure S5 Depletion of NAP1 does not affect cohesin complex stability. (**A**) Mass spectrometry analysis of SA IPed from either mock-treated or NAP1 knockdown (KD) cells. IPed SA was resolved by SDS-PAGE followed by colloidal blue staining (Figure 5E).The identity of the cohesin subunits were determined by mass spectrometric analysis and number of unique peptides (#) is shown. (**B**) NAP1 depletion does not affect PP2A levels. Immunoblotting analysis of S2 cell extracts prepared from mock-treated cells or cells depleted for NAP1 (KD), using the indicated antibodies. Histone H3 serves as a loading control.
(PDF)

Figure S6 NAP1 association with the core cohesin complex is cell-cycle regulated. Western blot analysis of SMC1 IPed from either mock-treated or NAP1 depleted (KD) cells treated (+) or untreated (−) with colhicine. The NAP1 association with cohesin is increased in colhicine treated cells, whereas PP2A binding is reduced in mitotically arrested cells. PP2A binding to cohesin is increased in NAP1 depleted cells and it remains bound to cohesin in mitotically arrested cells.
(PDF)

Figure S7 Effects of NAP1 and PP2A ectopic expression on mitosis. (**A**) Western blot analysis of S2 whole-cell extracts prepared from mock-treated cells or after knockdown of either NAP1, PP2A or both NAP1 and PP2A. Histone H3 serves as a loading control. (**B**) Western blot analysis of S2 whole-cell extracts prepared from cells transfected with constructs expressing either GFP or HA-tagged versions of NAP1, PP2A, both NAP1 and PP2A or a catalytically-inactive form of PP2A: PP2A^H59Q^. Ectopic over-expression (OE) of NAP1, PP2A and PP2A^H59Q^ was detected with antibodies against HA. Histone H3 serves as a loading control. (**C–D**) Analysis of mitotic chromosomes from colchicine-treated S2 cells after over-expression (OE) of GFP (Mock), NAP1, PP2A, both NAP1 and PP2A or the catalytic mutant PP2A^H59Q^. DNA visualized by DAPI staining is shown in red. Centromers are indicated by arrowheads, whereas loss of centromeric cohesion is indicated by full arrows. The localization of (**C′**) SA and (**D**) RAD21 (all shown in green) on mitotic chromosomes was determined by indirect immunofluorescence. The strong binding of SA and RAD21 to the arms of mitotic chromosomes was observed in ∼80% of cells that over-expressed PP2A, but not in mock-treated cells. (**E**) MeiS332 (green) accumulates on mitotic chromosomes of cells overexpressing PP2A (OE).
(PDF)

Table S1 Proteomics analysis of NAP1 and Cohesin protein interaction networks. **#** - number of unique peptides, score - Mascot score. The common contaminants, such as Hsc70, ribosomal proteins, etc. were excluded from the list.
(PDF)

Table S2 Proteomics analysis of PP2A protein interaction networks. **#** - number of unique peptides, score - Mascot score. The common contaminants, such as Hsc70, ribosomal proteins, etc. were excluded from the list.
(PDF)

Table S3 Sequences of the primers used for ChIP-qPCR.
(PDF)
