# Supplementary figures and images for: Histone Chaperone NAP1 Mediates Sister Chromatid Resolution by Counteracting Protein Phosphatase 2A
Source: PLoS Genet. 2013 Sep 26;9(9):e1003719. doi: 10.1371/journal.pgen.1003719 (PMC3784504; doi:10.1371/journal.pgen.1003719)

Figure S1

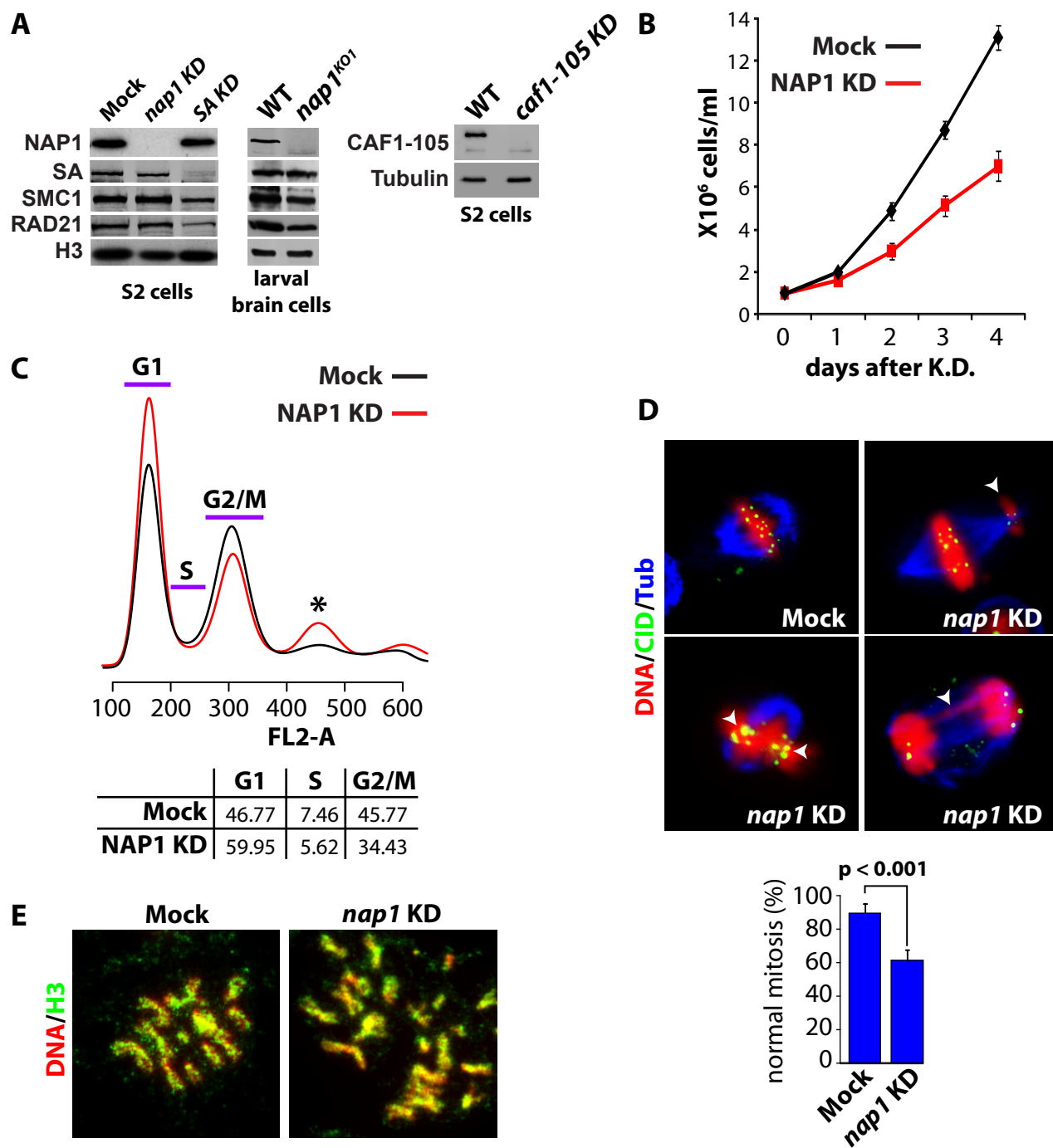

Supplement: Figure S1 — NAP1 is required for cell proliferation and normal mitosis. (A) Left panel: immunoblotting analysis of S2 whole-cell extracts prepared from mock-treated cells (Mock) or cells depleted for either NAP1 or SA by RNAi-mediated gene knockdown (KD). Histone H3 served as a loading control. Note that there is a modest reduction in SMC1 and RAD21 protein levels in cells depleted for SA. Middle panel: analysis of NAP1 and cohesin protein levels in Drosophila larvae brain cells homozygous for the NAP1 knockout allele nap1KO1 by immunoblotting with the indicated antibodies. Right panel: Western blot analysis of S2 whole-cell extracts prepared from mock-treated cells (Mock) or cells depleted for CAF1-105. (B) Proliferation of S2 cells treated with dsRNA directed against NAP1 is significantly reduced in comparison to the mock-treated cells. Cells were plated at 106 cells/ml, incubated with dsRNA directed against GFP (Mock) or NAP1 (KD) and counted for 4 consecutive days. Error bars indicate standard error of mean obtained from 3 different experiments. (C) Cell cycle profiles of S2 cells after depletion of NAP1. S2 cells were treated with dsRNA directed against NAP1 (KD) or GFP (Mock) for 2 days. Cell cycle profiles were determined by fluorescent assisted cell sorting (FACS) analysis. Cells were fixed, and DNA was stained with propidium iodide (PI). Quantification is based on gated cells. The percentage of cells in G1, S or G2/M phases and corresponding FACS profiles are shown for mock-treated cells (black) and NAP1 knockdown cells (NAP1 KD, red). Depletion of NAP1 caused a mild accumulation of the cells in G1 phase, and an increase of cells with a DNA content >4n (marked by asterisk). (D) NAP1 knockdown causes mitotic defects. Indirect immunofluorescent analysis of Drosophila S2 cells with antibodies against tubulin (blue) and CID (green) was performed to visualize mitotic spindles and centromeres, respectively. DNA was stained by DAPI (red). Representative mitotic defects in NAP [file pgen.1003719.s001.pdf]

Figure S2

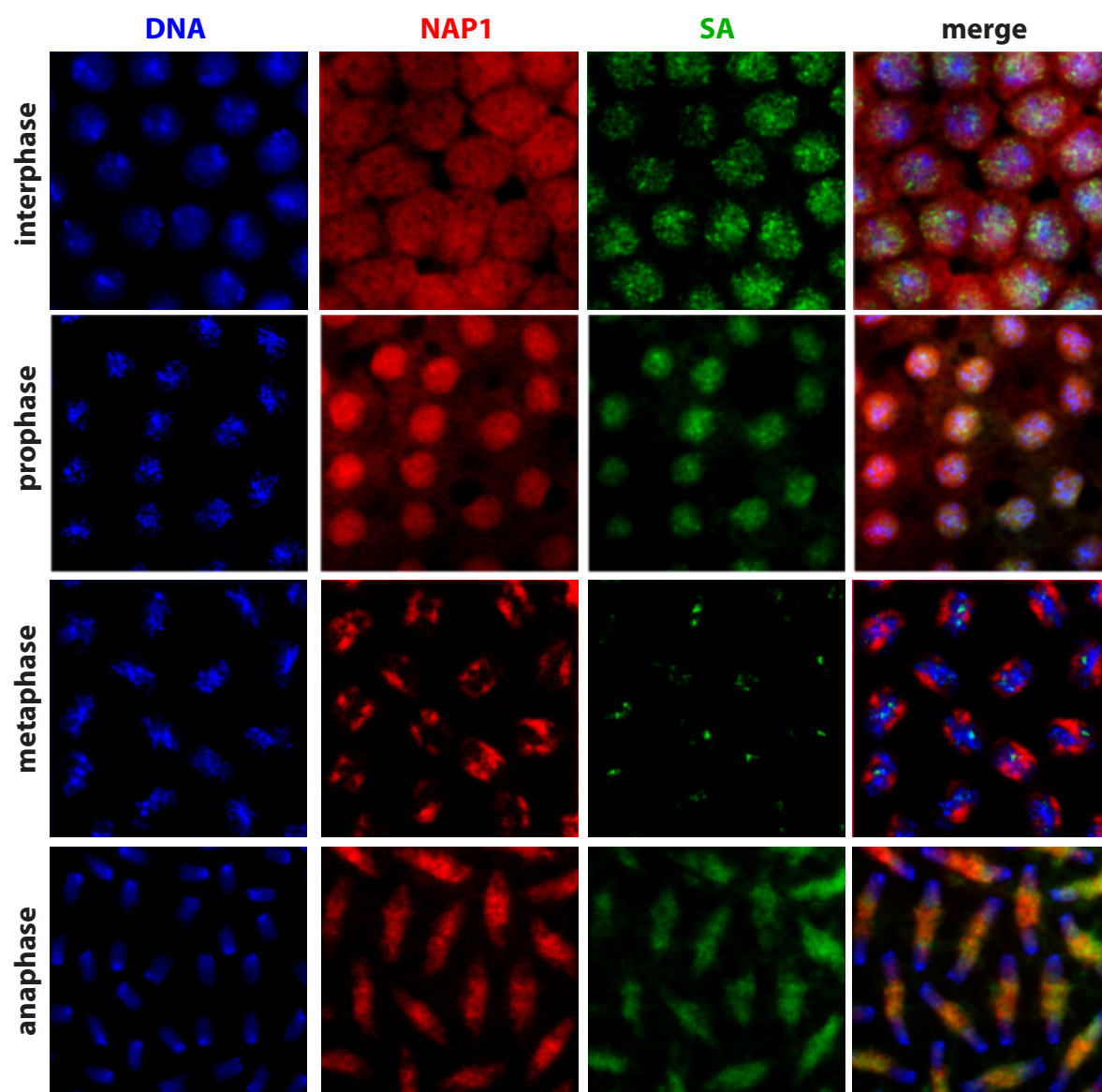

Supplement: Figure S2 — Changes in NAP1's subcellular localization during mitosis. Indirect immunofluorescence analysis of Drosophila embryos stained with antibodies against NAP1 (red) and SA (green). DNA was visualized by DAPI (blue). SA is nucleus during interphase and pro(meta)phase. By metaphase, the bulk of SA is removed from the chromosome arms, but by anaphase it binds the chromosomes again. During interphase, NAP1 is distributed roughly equally between nucleus and cytoplasm. However, there is a strong increase in NAP1 nuclear localization at pro(meta)phase. During metaphase, NAP1 dissociates from the DNA, but still surrounds the mitotic chromosomes. During anaphase, NAP1 starts to re-associate with the chromosomes. (PDF) [file pgen.1003719.s002.pdf]

Figure S3

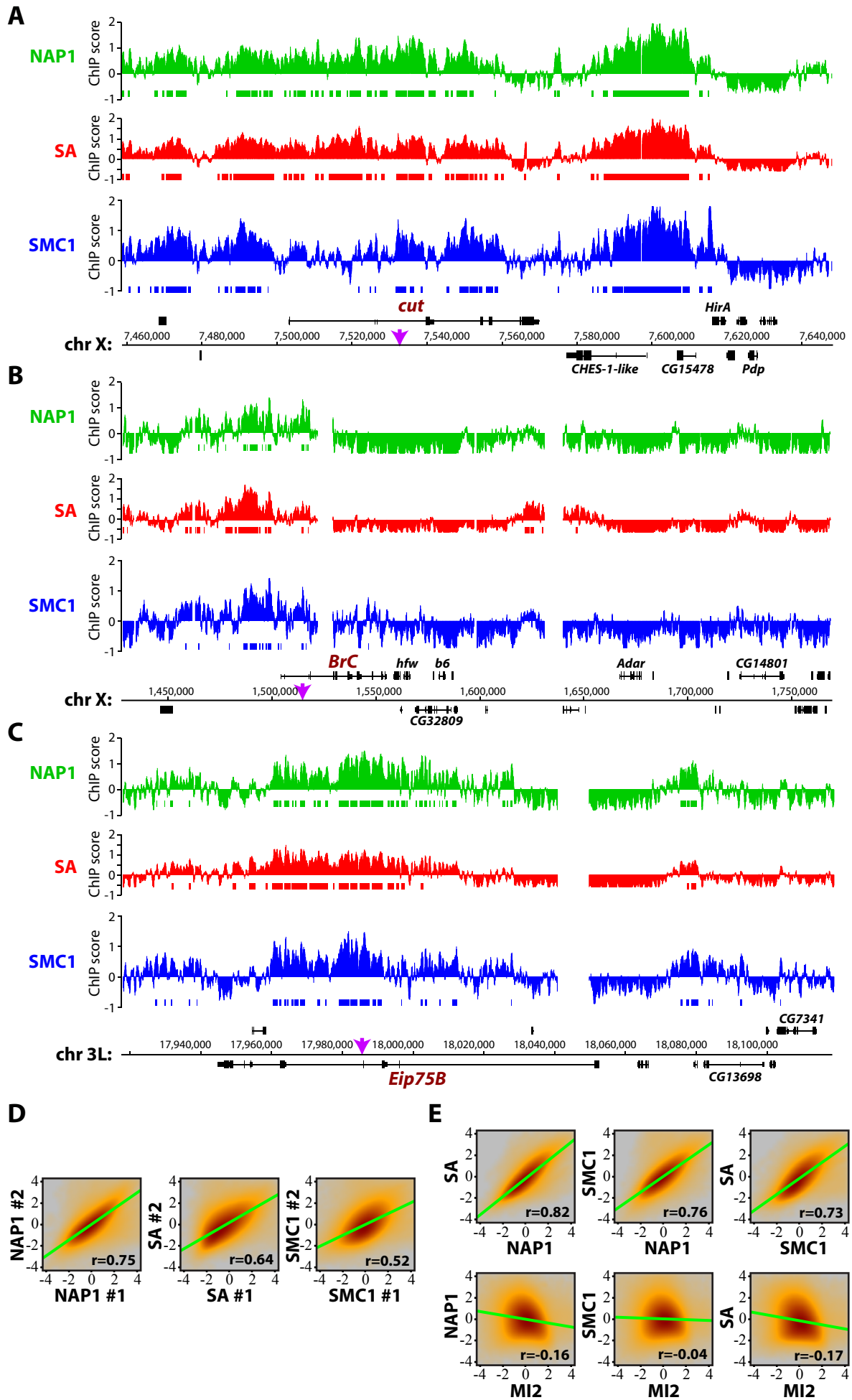

Supplement: Figure S3 — Genome-wide binding profiling of NAP1 and cohesin by ChIP-chip. (A–C) Genomic view of NAP1 (green), SA (red) and SMC1 (blue) ChIP-chip enrichment profiles across genomic regions harboring the cut NOTCH target gene (A), and two ecdysone-inducible loci harboring Broad Complex (BrC) (B) and Eip75B (C) genes. Filtered binding sites are indicated as bars below the respective profiles. ChIP-chip enrichment scores, genomic coordinates and genes are indicated. Regions examined by ChIP-qPCR are indicated by arrows. (D) The genome-wide ChIP-chip profiles of NAP1 and cohesin subunits SMC1 and SA are highly correlated between independent biological replicates. (E) The genome-wide ChIP-chip profiles of NAP1 and cohesin subunits SMC1 and SA are highly correlated to each other, in contrast to the binding profile of the ATP-dependent chromatin remodeler MI2. Pairwise smooth scatter plots of averaged NAP1, SA, SMC1 and MI2 ChIP-chip enrichment scores are shown, and correlations (r) and linear regression lines are indicated. (PDF) [file pgen.1003719.s003.pdf]

**Figure S4**

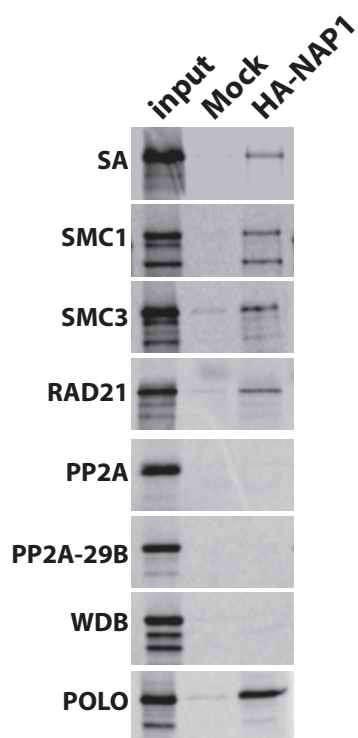

Supplement: Figure S4 — NAP1 interacts in vitro with cohesin subunits, but not PP2A. Protein-protein interaction assay using recombinant HA-tagged NAP1, expressed in Sf9 cells using the baculovirus system (Figure 5A). Cohesin subunits (SA, SMC1, SMC3 and RAD21), PP2A catalytic subunit (PP2A) and regulatory subunits (PP2A-29B, WDB), and Polo like kinase were produced using a coupled in vitro transcription/translation (IVT) system in the presence of [35S]methionine. Radiolabeled proteins were incubated with Protein A beads decorated with anti-HA antibodies bound to HA-NAP1 or lacking HA-NAP1 (Mock). Following extensive washes with a buffer containing 600 mM KCl and 0.1% NP40, proteins were resolved by sodium dodecyl sulfate polyacrylamide gel electrophoresis (SDS-PAGE) and detected by autoradiography. Input corresponds to 10% of the binding reaction. (PDF) [file pgen.1003719.s004.pdf]

**A**

| SA IP               |    |    |
|---------------------|----|----|
| Mock <i>nap1</i> KD |    |    |
| SMC1                | 68 | 70 |
| SMC3                | 76 | 78 |
| SA                  | 66 | 63 |
| RAD21               | 34 | 32 |
| # of peptides       |    |    |

**B**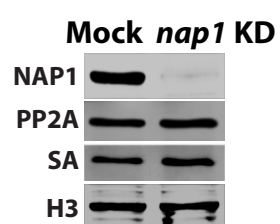

Supplement: Figure S5 — Depletion of NAP1 does not affect cohesin complex stability. (A) Mass spectrometry analysis of SA IPed from either mock-treated or NAP1 knockdown (KD) cells. IPed SA was resolved by SDS-PAGE followed by colloidal blue staining (Figure 5E).The identity of the cohesin subunits were determined by mass spectrometric analysis and number of unique peptides (#) is shown. (B) NAP1 depletion does not affect PP2A levels. Immunoblotting analysis of S2 cell extracts prepared from mock-treated cells or cells depleted for NAP1 (KD), using the indicated antibodies. Histone H3 serves as a loading control. (PDF) [file pgen.1003719.s005.pdf]

**Figure S6**

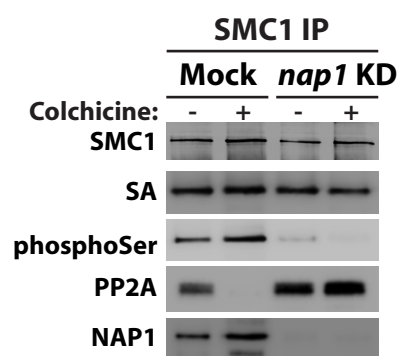

Supplement: Figure S6 — NAP1 association with the core cohesin complex is cell-cycle regulated. Western blot analysis of SMC1 IPed from either mock-treated or NAP1 depleted (KD) cells treated (+) or untreated (−) with colhicine. The NAP1 association with cohesin is increased in colhicine treated cells, whereas PP2A binding is reduced in mitotically arrested cells. PP2A binding to cohesin is increased in NAP1 depleted cells and it remains bound to cohesin in mitotically arrested cells. (PDF) [file pgen.1003719.s006.pdf]

Figure S7

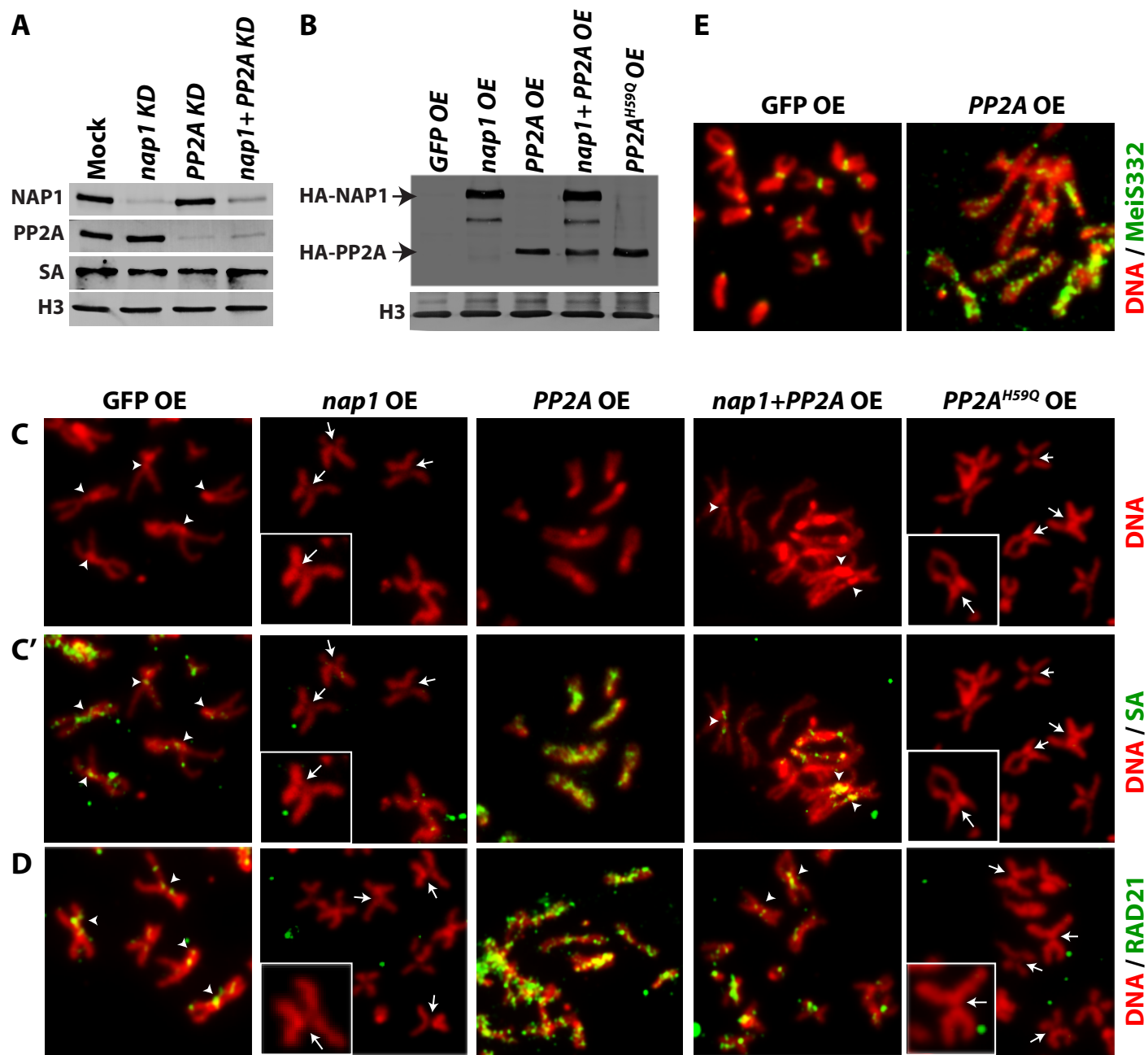

Supplement: Figure S7 — Effects of NAP1 and PP2A ectopic expression on mitosis. (A) Western blot analysis of S2 whole-cell extracts prepared from mock-treated cells or after knockdown of either NAP1, PP2A or both NAP1 and PP2A. Histone H3 serves as a loading control. (B) Western blot analysis of S2 whole-cell extracts prepared from cells transfected with constructs expressing either GFP or HA-tagged versions of NAP1, PP2A, both NAP1 and PP2A or a catalytically-inactive form of PP2A: PP2AH59Q. Ectopic over-expression (OE) of NAP1, PP2A and PP2AH59Q was detected with antibodies against HA. Histone H3 serves as a loading control. (C–D) Analysis of mitotic chromosomes from colchicine-treated S2 cells after over-expression (OE) of GFP (Mock), NAP1, PP2A, both NAP1 and PP2A or the catalytic mutant PP2AH59Q. DNA visualized by DAPI staining is shown in red. Centromers are indicated by arrowheads, whereas loss of centromeric cohesion is indicated by full arrows. The localization of (C′) SA and (D) RAD21 (all shown in green) on mitotic chromosomes was determined by indirect immunofluorescence. The strong binding of SA and RAD21 to the arms of mitotic chromosomes was observed in ∼80% of cells that over-expressed PP2A, but not in mock-treated cells. (E) MeiS332 (green) accumulates on mitotic chromosomes of cells overexpressing PP2A (OE). (PDF) [file pgen.1003719.s007.pdf]
