# Supplementary material for: Histone Chaperone NAP1 Mediates Sister Chromatid Resolution by Counteracting Protein Phosphatase 2A
Source: PLoS Genet. 2013 Sep 26;9(9):e1003719. doi: 10.1371/journal.pgen.1003719 (PMC3784504; doi:10.1371/journal.pgen.1003719)
Supplement: Table S1 — Proteomics analysis of NAP1 and Cohesin protein interaction networks. # - number of unique peptides, score - Mascot score. The common contaminants, such as Hsc70, ribosomal proteins, etc. were excluded from the list. (PDF) [file pgen.1003719.s008.pdf]

Table S1

FlyBase	Symbol	Comments	MW (Da)	embryo NAP1 Ab1 #	embryo NAP1 Ab1 score	embryo NAP1 Ab2 #	embryo NAP1 Ab2 score	S2 cells NAP1 Ab1 #	S2 cells NAP1 Ab1 score	embryo SA #	embryo SA score	embryo SMC1 #	embryo SMC1 score
FBgn0015268	**Nap1**	**H2A/H2B histone chaperone**	42782	24	1979	18	1379	15	902	4	177	2	102
FBgn0040283	**SMC1**	**Cohesin subunit**	142881	19	1184	15	866	30	1952	91	5804	64	5354
FBgn0015615	**Cap**	**Cohesin subunit SMC3**	140036	31	2184	24	1721	24	1747	97	6505	70	5710
FBgn0260987	**RAD21**	**Cohesin subunit**	79945	5	250	5	228	14	872	34	2187	32	2515
FBgn0020616	**SA**	**Cohesin subunit**	130116	14	1053	5	377	18	1225	67	4334	54	3899
FBgn0004177	**Mts**	**Protein phosphatase 2A**	35469	6	403	3	200	3	217	5	328	4	277
FBgn0003124	**polo**	**PLK kinase**	66974	7	476	2	106	3	153	4	195	1	41
FBgn0259785	**pzg**	**CTCF/BORIS homolog**	105081	8	584	9	697	13	1054	17	725	14	1034
FBgn0033890	**CG13350**	**Cohesin loading CTF4**	96647	24	1678	27	1895	27	1938	0	0	0	0
FBgn0026401	**NipB**	**Cohesin loading**	233824	0	0	0	0	0	0	23	755	4	340
FBgn0038300	**CG4203**	**Cohesin loading MAU2**	71217	0	0	0	0	0	0	12	405	4	192
FBgn0004913	Gnf1	RFC subunit	108615	9	580	5	358	2	205	16	697	2	197
FBgn0030871	CG8142	RFC subunit	39555	5	311	5	331	6	417	13	629	5	415
FBgn0260985	RfC4	RFC subunit	37173	9	580	7	426	6	454	12	568	7	501
FBgn0028700	RfC38	RFC subunit	45504	10	693	10	581	10	713	17	843	8	479
FBgn0032244	RfC3	RFC subunit	37408	14	979	11	886	8	580	14	830	10	797
FBgn0022764	Sin3A	RLAF subunit	220516	35	2589	27	1933	55	4451	28	1062	12	662
FBgn0015805	Rpd3	RLAF subunit	58331	14	1029	12	804	18	1367	9	455	8	473
FBgn0031759	LID	RLAF subunit	203993	46	3694	41	2912	57	4350	0	0	0	0
FBgn0031377	EMSY	RLAF subunit	108136	23	1642	16	1142	33	2736	0	0	0	0
FBgn0029861	PF1	RLAF subunit	98336	18	1204	16	1064	25	1747	0	0	0	0
FBgn0027378	MRG15	RLAF subunit	47194	11	789	11	849	10	851	0	0	0	0

FlyBase	Symbol	Comments	MW (Da)	embryo				S2 cells		embryo			
				NAP1 Ab1		NAP1 Ab2		NAP1 Ab1		SA		SMC1	
				#	score	#	score	#	score	#	score	#	score
FBgn0014861	Mcm2	MCM subunit	100415	25	1973	20	1600	22	1723	0	0	0	0
FBgn0015929	dpa	MCM subunit MCM4	96610	37	2890	34	2743	37	2997	0	0	0	0
FBgn0025815	Mcm6	MCM subunit	92353	21	1636	25	1816	21	1296	0	0	0	0
FBgn0037382	Hpr1	THO subunit	80760	14	939	12	761	25	1981	0	0	0	0
FBgn0031390	tho2	THO subunit	188608	17	1181	10	639	44	3316	0	0	0	0
FBgn0037569	tex	THO subunit	36406	8	556	5	332	15	1209	0	0	0	0
FBgn0034939	thoc5	THO subunit	70889	8	445	6	334	13	1042	0	0	0	0
FBgn0036263	thoc6	THO subunit	39221	4	284	1	88	9	593	0	0	0	0
FBgn0035110	thoc7	THO subunit	33052	6	449	4	249	9	714	0	0	0	0
FBgn0025634	CG13367	Transcription factor GATAD1	43940	8	634	7	553	10	774	0	0	0	0
FBgn0004856	Bx42	Transcriptional regulator	61157	13	1005	9	671	18	1356	0	0	0	0
FBgn0003607	Su(var)205	Chromatin silencing	23185	7	665	7	593	7	638	0	0	0	0
FBgn0030082	HP1b	Chromatin silencing	25974	7	570	7	517	6	444	0	0	0	0
FBgn0032475	Sfmbt	Chromatin silencing	133666	15	905	11	714	16	963	0	0	0	0
FBgn0020496	CtBP	Co-repressor	42252	7	566	6	296	13	1147	0	0	0	0
FBgn0040465	Dip3	Transcriptional regulator	39737	13	1148	12	962	2	123	0	0	0	0
FBgn0014269	prod	Transcriptional regulator	39428	9	620	5	293	15	1277	0	0	0	0
FBgn0026575	hang	Transcriptional regulator	209717	3	178	3	162	19	1217	0	0	0	0
FBgn0033607	CG9062	WDR48 homolog	75266	8	566	5	269	20	1690	0	0	0	0
FBgn0011802	Gem3	Dxd20 homolog	116508	28	2173	24	1803	1	51	0	0	0	0
FBgn0259483	Mob4	Phocein homolog	25747	7	617	7	570	7	528	0	0	0	0
FBgn0034987	CG3363	-	241964	64	4511	46	2803	51	3486	0	0	0	0
FBgn0029686	CG2941	-	105015	26	1944	26	1795	4	315	0	0	0	0
FBgn0036624	CG4877	-	131942	24	1544	13	771	11	564	0	0	0	0

Table S1

FlyBase	Symbol	Comments	MW (Da)	embryo NAP1 Ab1 #	embryo NAP1 Ab1 score	embryo NAP1 Ab2 #	embryo NAP1 Ab2 score	S2 cells NAP1 Ab1 #	S2 cells NAP1 Ab1 score	embryo SA #	embryo SA score	embryo SMC1 #	embryo SMC1 score
FBgn0033998	row	Transcriptional regulator	143681	0	0	0	0	11	886	11	416	4	250
FBgn0001994	crp	Transcription factor AP-4	67163	0	0	0	0	13	898	9	433	13	759
FBgn0039227	polybromo	PBAP subunit	189694	0	0	0	0	0	0	49	2263	9	535
FBgn0042085	Bap170	PBAP subunit	183029	0	0	0	0	0	0	28	1285	4	213
FBgn0087008	e(y)3/SAYP	PBAP subunit	212726	0	0	0	0	0	0	10	466	0	0
FBgn0031947	CG7154/BRD7	PBAP subunit	95922	0	0	0	0	0	0	5	191	1	49
FBgn0000212	brm	PBAP subunit	185089	0	0	0	0	0	0	29	1108	10	547
FBgn0002783	mor	PBAP subunit	131361	0	0	0	0	0	0	23	1242	12	716
FBgn0030093	dalao	PBAP subunit	78812	0	0	0	0	0	0	13	522	5	421
FBgn0025463	Bap60	PBAP subunit	58170	0	0	0	0	0	0	17	851	7	410
FBgn0025716	Bap55	PBAP subunit	47319	0	0	0	0	0	0	12	699	8	573
FBgn0011715	Snr1	PBAP subunit	41911	0	0	0	0	0	0	8	321	7	476
FBgn0260962	pic/DDB1	DDB1-CUL4 subunit	126045	0	0	0	0	0	0	13	452	20	1274
FBgn0033260	Cul-4	DDB1-CUL4 subunit	94408	0	0	0	0	0	0	9	361	21	1370
FBgn0027948	msps	TACC-MSPS subunit	226844	0	0	0	0	0	0	13	423	33	1889
FBgn0026620	tacc	TACC-MSPS subunit	143966	0	0	0	0	0	0	4	128	21	1342
