# Supplementary material for: Histone Chaperone NAP1 Mediates Sister Chromatid Resolution by Counteracting Protein Phosphatase 2A
Source: PLoS Genet. 2013 Sep 26;9(9):e1003719. doi: 10.1371/journal.pgen.1003719 (PMC3784504; doi:10.1371/journal.pgen.1003719)
Supplement: Table S2 — Proteomics analysis of PP2A protein interaction networks. # - number of unique peptides, score - Mascot score. The common contaminants, such as Hsc70, ribosomal proteins, etc. were excluded from the list. (PDF) [file pgen.1003719.s009.pdf]

Table S2

FlyBase	Symbol	Comments	MW (Da)	#	score
FBgn0004177	**mts/PP2A**	**PP2A catalytic**	35469	14	969
FBgn0260439	**Pp2A-29B**	**PP2A regulator**	65424	17	1165
FBgn0027492	**wdb**	**PP2A regulator**	59726	2	87
FBgn0015268	**Nap1**	**Nap1**	42768	7	355
FBgn0020616	**SA**	**Cohesin subunit**	130116	8	397
FBgn0040283	**SMC1**	**Cohesin subunit**	142881	7	384
FBgn0015615	**Cap**	**Cohesin subunit SMC3**	140036	8	456
FBgn0260987	**RAD21**	**Cohesin subunit**	79945	3	123
FBgn0026401	**NipB**	**Cohesin loading**	233883	12	581
FBgn0038300	**CG4203**	**Cohesin loading MAU2**	71217	6	232
FBgn0031759	LID	RLAF subunit	203993	6	356
FBgn0022764	Sin3A	RLAF subunit	220516	23	1179
FBgn0029861	PF1	RLAF subunit	98336	4	165
FBgn0015805	Rpd3	RLAF subunit	58331	14	890
FBgn0027378	MRG15	RLAF subunit	47194	8	515
FBgn0004103	Pp1-87B	PP1 catalytic	34542	8	537
FBgn0003134	Pp1alpha-96A	PP1 catalytic	37370	7	472
FBgn0000711	flw	PP1 catalytic	37740	4	206
FBgn0023177	Pp4-19C	PP3 catalytic	35341	5	352
FBgn0030208	PPP4R2r	PP4 catalytic	66809	7	407
FBgn0005777	PpD3	PP5 catalytic	59264	1	40
FBgn0003139	PpV	PP6 catalytic	34759	2	61
FBgn0003124	polo	S/T kinase	66974	15	714
FBgn0024227	ial/AurB	S/T kinase	38301	10	510
FBgn0004367	mei-41	ATR kinase	289330	10	466
FBgn0045035	tefu	ATM kinase	317991	5	270
FBgn0086899	tlk	S/T kinase	135552	9	554
FBgn0015024	CkIalpha	Casein kinase	39535	10	539
FBgn0264492	CkIIalpha	Casein kinase	39960	14	744
FBgn0000259	CkIIbeta	Casein kinase	24829	5	255
FBgn0003178	PyK	Pyruvate kinase	57440	15	827
FBgn0016696	Pitslre/cdk11A	S/T kinase	108838	18	813
FBgn0015618	Cdk8	S/T kinase	53682	5	213
FBgn0027889	ball	VR kinase	65994	8	400

Table S2

FlyBase	Symbol	Comments	MW (Da)	#	score
FBgn0002069	Aats-asp	Aminoacyl-tRNA synthesis	59059	17	1002
FBgn0027084	Aats-lys	Aminoacyl-tRNA synthesis	64661	12	638
FBgn0027093	Aats-arg	Aminoacyl-tRNA synthesis	75577	14	728
FBgn0027086	Aats-ile	Aminoacyl-tRNA synthesis	141099	25	1185
FBgn0033351	CG8235	Aminoacyl-tRNA synthesis	34401	6	253
FBgn0036515	CG12304	Aminoacyl-tRNA synthesis	33242	5	214
FBgn0034401	CG15100	Aminoacyl-tRNA synthesis	112484	13	816
FBgn0053123	CG33123	Aminoacyl-tRNA synthesis	134873	15	820
FBgn0005674	Aats-glupro	Aminoacyl-tRNA synthesis	189412	19	1041
FBgn0027090	Aats-gln	Aminoacyl-tRNA synthesis	87508	7	306
FBgn0027081	Aats-thr	Aminoacyl-tRNA synthesis	79345	6	277
FBgn0051739	mdy	Aminoacyl-tRNA synthesis	121520	9	484
FBgn0025457	Bub3	cell cycle	37415	6	397
FBgn0026326	Mad1	cell cycle	85018	14	752
FBgn0020407	Mat89Bb	cell cycle	75728	11	691
FBgn0027783	SMC2	Condensin	134335	15	860
FBgn0014127	barr	Condensin	82762	8	393
FBgn0039680	CAP-D2	Condensin	157500	11	430
FBgn0015391	glu	Condensin	159883	10	499
FBgn0259876	Cap-G	Condensin	153878	6	246
FBgn0003732	Top2	DNA topoisomerase 2	164396	47	2710
FBgn0010278	Ssrp	FACT complex	81533	12	613
FBgn0002183	dre4	FACT complex	123583	18	936
FBgn0040273	Spt5	FACT complex	119445	5	283
FBgn0039691	IntS11	Integrator complex	67592	15	879
FBgn0026679	IntS4	Integrator complex	113423	21	1207
FBgn0036038	defl	Integrator complex	112288	19	1072
FBgn0036570	IntS9	Integrator complex	73272	11	632
FBgn0034964	IntS1	Integrator complex	235099	37	2126
FBgn0038168	omd	Integrator complex	112972	16	961
FBgn0030858	IntS2	Integrator complex	124890	15	838
FBgn0261383	IntS6	Integrator complex	137108	18	1018
FBgn0025830	IntS8	Integrator complex	113480	13	735
FBgn0262117	IntS3	Integrator complex	123951	14	670
FBgn0035462	IntS10	Integrator complex	72257	5	303
FBgn0011604	Iswi	ISWI remodeler	118873	31	1557
FBgn0034503	MED8	MEDIATOR	27948	6	365
FBgn0004597	CycC	MEDIATOR	31292	5	267
FBgn0038578	MED17	MEDIATOR	71557	11	565
FBgn0037359	MED27	MEDIATOR	33868	5	256

FlyBase	Symbol	Comments	MW (Da)	#	score
FBgn0037024	CG4365	metabolism	34184	12	649
FBgn0000150	awd	metabolism	17170	5	288
FBgn0000064	Ald	metabolism	39047	12	752
FBgn0000055	Adh	metabolism	27761	7	388
FBgn0043456	CG4747	metabolism	65252	15	953
FBgn0032350	CG6287	metabolism	35235	9	586
FBgn0035811	CG12262	metabolism	45871	10	550
FBgn0027588	CG14476	metabolism	105731	24	1134
FBgn0001092	Gapdh2	metabolism	35369	7	471
FBgn0001091	Gapdh1	metabolism	35350	7	447
FBgn0033879	CG6543	metabolism	31582	6	303
FBgn0027291	l(1)G0156	metabolism	40844	6	318
FBgn0036030	CG6767	metabolism	42696	6	373
FBgn0033160	CG11107	miRNA processing	82652	12	610
FBgn0015075	Ddx1	miRNA processing	80869	5	240
FBgn0087035	AGO2	miRNA processing	136850	8	354
FBgn0000253	Cam	NA	16811	8	496
FBgn0013269	FK506-bp1	NA	39344	16	1002
FBgn0032105	borr	NA	34681	13	888
FBgn0035541	CG15019	NA	17274	6	321
FBgn0086768	Pcmt	NA	24590	9	608
FBgn0032731	CG10641	NA	25087	10	597
FBgn0250837	dUTPase	NA	19959	9	480
FBgn0051852	Tap42	NA	43319	14	821
FBgn0038830	CG17272	NA	17135	5	332
FBgn0015379	dod	NA	18377	6	466
FBgn0037746	CG8478	NA	63116	15	855
FBgn0035059	CG3894	NA	35531	9	601
FBgn0004432	Cyp1	NA	24666	6	369
FBgn0260991	Incenp	NA	83537	20	1292
FBgn0034313	CG5726	NA	86592	19	1327
FBgn0039743	CG7946	NA	52839	14	683
FBgn0261524	lic	NA	38195	11	487
FBgn0039274	CG11920	NA	34667	8	366
FBgn0050390	Sgf29	NA	32116	6	373
FBgn0027066	Eb1	NA	32576	7	406
FBgn0039868	CG11563	NA	24754	6	332
FBgn0025366	Ip259	NA	30029	7	392
FBgn0014133	bif	NA	130091	27	1726
FBgn0003187	qua	NA	100960	22	1260
FBgn0004401	Pep	NA	78048	14	891
FBgn0037670	CG8436	NA	27717	5	351

FlyBase	Symbol	Comments	MW (Da)	#	score
FBgn0027514	CG1024	NA	62488	12	655
FBgn0015527	pen	NA	81302	14	764
FBgn0033951	CG10139	NA	25513	5	276
FBgn0033059	CG7845	NA	46674	8	522
FBgn0033185	CG1603	NA	68326	12	648
FBgn0042134	CG18811	NA	103590	17	1267
FBgn0038476	kuk	NA	60160	11	631
FBgn0259220	Doa	NA	67894	10	519
FBgn0037261	CG9775	NA	46128	6	404
FBgn0037470	CG1091	NA	63945	10	555
FBgn0030959	CG6961	NA	53015	9	483
FBgn0033741	CG8545	NA	99841	17	889
FBgn0020392	Nmt	NA	53833	9	446
FBgn0004587	B52	NA	42813	7	492
FBgn0031070	CG12702	NA	97173	15	869
FBgn0022349	CG1910	NA	49324	7	453
FBgn0023514	CG14805	NA	55949	9	543
FBgn0036248	ssp	NA	43342	7	295
FBgn0039929	CG11076	NA	31841	5	248
FBgn0038746	Surf6	NA	38135	5	285
FBgn0030699	CG8578	NA	45402	7	483
FBgn0037504	CG1142	NA	32696	5	231
FBgn0020279	lig	NA	134561	13	1136
FBgn0259173	corn	NA	121987	17	960
FBgn0037811	CG12592	NA	75699	11	519
FBgn0036886	CG9300	NA	76358	11	638
FBgn0001961	Sop2	NA	41601	6	260
FBgn0032859	Arc-p34	NA	35104	5	223
FBgn0037621	CG9797	NA	48093	6	245
FBgn0037878	CG6693	NA	34890	5	284
FBgn0039627	CG11837	NA	35048	5	256
FBgn0259745	wech	NA	90573	13	608
FBgn0032454	CG5787	NA	100258	14	693
FBgn0037810	sle	NA	158654	22	1197
FBgn0030293	CG1737	NA	102112	14	920
FBgn0033454	CG1671	NA	88537	12	658
FBgn0011335	l(3)j2D3	NA	44830	5	250
FBgn0010770	ppan	NA	53093	7	346
FBgn0053505	U3-55K	NA	53286	7	305
FBgn0036184	PCID2	NA	45180	6	268
FBgn0031575	Cep97	NA	92644	12	644
FBgn0052344	CG6994	NA	93370	12	605

Table S2

FlyBase	Symbol	Comments	MW (Da)	#	score
FBgn0014163	fax	NA	46644	6	376
FBgn0037489	CG1234	NA	94492	12	649
FBgn0025633	CG13366	NA	121654	15	929
FBgn0030061	CG1785	NA	55788	7	267
FBgn0031314	CG4785	NA	65049	8	474
FBgn0030067	Rbm13	NA	40586	5	313
FBgn0005655	mus209	PCNA	28830	5	342
FBgn0003607	Su(var)205	Transcription/chromatin	23185	7	467
FBgn0040078	pont	Transcription/chromatin	50242	18	1104
FBgn0015610	Caf1	Transcription/chromatin	48635	10	696
FBgn0040075	rept	Transcription/chromatin	53541	15	945
FBgn0035121	Tudor-SN	Transcription/chromatin	103100	30	1725
FBgn0259784	Det	Transcription/chromatin	17455	5	293
FBgn0002780	mod	Transcription/chromatin	60310	12	862
FBgn0030082	HP1b	Transcription/chromatin	25974	6	219
FBgn0039904	Hcf	Transcription/chromatin	160185	33	1730
FBgn0032030	CG17293	Transcription/chromatin	35355	7	330
FBgn0027524	CG3909	Transcription/chromatin	36100	7	383
FBgn0035624	Eaf6	Transcription/chromatin	24400	5	261
FBgn0031769	CG9135	Transcription/chromatin	53460	10	543
FBgn0029704	CG2982	Transcription/chromatin	73098	13	689
FBgn0027951	MTA1-like	Transcription/chromatin	96609	17	852
FBgn0039459	CG5491	Transcription/chromatin	34334	5	279
FBgn0030434	CG4400	Transcription/chromatin	29560	5	223
FBgn0037583	CG9684	Transcription/chromatin	71908	12	632
FBgn0037657	hyx	Transcription/chromatin	61385	11	572
FBgn0031711	CG6907	Transcription/chromatin	48712	8	508
FBgn0027835	Dp1	Transcription/chromatin	144276	22	1235
FBgn0000283	Cp190	Transcription/chromatin	121679	16	957
FBgn0011785	BRWD3	Transcription/chromatin	248662	33	1726
FBgn0037624	CG8223	Transcription/chromatin	51890	7	500
FBgn0014269	prod	Transcription/chromatin	39428	5	296
FBgn0015664	Dref	Transcription/chromatin	80727	10	555
FBgn0028700	RfC38	RF-C complex	40815	12	788
FBgn0032244	RfC3	RF-C complex	37408	10	674
FBgn0260985	RfC4	RF-C complex	37173	9	507
FBgn0030871	CG8142	RF-C complex	39555	8	381
FBgn0031657	CG3756	RNA polI	38138	6	285
FBgn0003278	RpI135	RNA polI	128444	9	462
FBgn0019938	RpI1	RNA polI	185410	6	322
FBgn0262955	RpII140	RNA polII	134043	7	375
FBgn0003277	RpII215	RNA polII	209168	5	185

Table S2

FlyBase	Symbol	Comments	MW (Da)	#	score
FBgn0040284	SF2	RNA processing	28350	15	887
FBgn0003261	Rm62	RNA processing	62474	29	1704
FBgn0011692	pav	RNA processing	100667	32	2301
FBgn0261789	SmD2	RNA processing	13504	5	276
FBgn0261119	Prp19	RNA processing	55199	15	948
FBgn0261619	pAbp	RNA processing	69925	22	1439
FBgn0086897	sqd	RNA processing	36184	11	760
FBgn0261014	TER94	RNA processing	88859	24	1581
FBgn0034921	Dcp1	RNA processing	41359	11	697
FBgn0086356	tum	RNA processing	69763	19	1304
FBgn0010774	Aly	RNA processing	27852	7	401
FBgn0262601	SmB	RNA processing	21021	6	303
FBgn0035720	CG10077	RNA processing	88210	22	1376
FBgn0037573	eIF4AIII	RNA processing	45645	10	658
FBgn0031229	CG3436	RNA processing	38860	10	452
FBgn0000171	bel	RNA processing	85081	19	1234
FBgn0015331	abs	RNA processing	69488	16	981
FBgn0014189	Hel25E	RNA processing	48652	10	520
FBgn0035987	CG3689	RNA processing	26962	6	427
FBgn0039558	CG4980	RNA processing	31342	7	353
FBgn0260010	rump	RNA processing	66731	12	699
FBgn0036735	Edc3	RNA processing	73415	15	845
FBgn0035136	CG6905	RNA processing	93085	18	966
FBgn0022942	Cbp80	RNA processing	93227	15	689
FBgn0039566	CG4849	RNA processing	110650	21	1238
FBgn0032487	Ski6	RNA processing	27022	5	279
FBgn0034879	Rrp4	RNA processing	33624	6	309
FBgn0014366	noi	RNA processing	58422	10	470
FBgn0033688	Prp8	RNA processing	279578	46	2198
FBgn0037737	Pnn	RNA processing	36380	6	364
FBgn0017457	U2af38	RNA processing	29877	5	193
FBgn0036548	CG5931	RNA processing	244510	37	1870
FBgn0037550	CG9667	RNA processing	31352	5	282
FBgn0004419	me31B	RNA processing	51945	8	415
FBgn0000662	fl(2)d	RNA processing	59268	9	457
FBgn0039867	CstF-50	RNA processing	46922	7	310
FBgn0028382	cyp33	RNA processing	33276	5	230
FBgn0035162	CG13900-RA	RNA processing	136616	19	1070
FBgn0263198	Acn	RNA processing	83720	12	867
FBgn0027873	Cpsf100	RNA processing	85418	12	575
FBgn0022959	yps	RNA processing	38181	5	248
FBgn0262734	Rbp2	RNA processing	38263	5	334

Table S2

FlyBase	Symbol	Comments	MW (Da)	#	score
FBgn0005411	U2af50	RNA processing	46655	6	333
FBgn0027841	CstF-64	RNA processing	46185	6	271
FBgn0032340	Ge-1	RNA processing	149251	19	1117
FBgn0027548	nito	RNA processing	89053	11	452
FBgn0029979	CG10777	RNA processing	100418	11	541
FBgn0036340	SRm160	RNA processing	107593	9	616
FBgn0031883	CG11266	RNA processing	66482	8	556
FBgn0025463	Bap60	SWI-SNF complexes	58170	21	1186
FBgn0025716	Bap55	SWI-SNF complexes	47319	13	765
FBgn0030093	dalao	SWI-SNF complexes	78672	17	976
FBgn0002783	mor	SWI-SNF complexes	131361	24	1578
FBgn0000212	brm	SWI-SNF complexes	185089	33	1920
FBgn0011715	Snr1	SWI-SNF complexes	41911	6	395
FBgn0039227	polybromo	SWI-SNF complexes	189694	25	1409
FBgn0042085	Bap170	SWI-SNF complexes	183029	13	741
FBgn0261885	osa	SWI-SNF complexes	284064	13	682
FBgn0027568	Cand1	Ubiquitin E3 ligase complex	139355	6	282
FBgn0260962	pic	Ubiquitin E3 ligase complex	126045	21	1105
FBgn0033260	Cul-4	Ubiquitin E3 ligase complex	94408	19	924
