# Supplementary material for: Histone Chaperone NAP1 Mediates Sister Chromatid Resolution by Counteracting Protein Phosphatase 2A
Source: PLoS Genet. 2013 Sep 26;9(9):e1003719. doi: 10.1371/journal.pgen.1003719 (PMC3784504; doi:10.1371/journal.pgen.1003719)
Supplement: Table S3 — Sequences of the primers used for ChIP-qPCR. (PDF) [file pgen.1003719.s010.pdf]

Table S3

**gene**	**region (chr.: position bp)**	**forward**	**reverse**
HLHmα*-m*β	3R: 21,832,944-21,833,048	GCCGGCGACGATGACGCTCGC	TCGAGTAGCTGCCGTCTCGGAGCT
HLHm5-m6	3R: 21,856,326-21,856,404	ACCGCTCGAGACGCGTCGTGT	CGCTGGCTTTCCGTGTATCCGTGCC
HLHm7	3R: 21,862,621-21,862,714	TGCTCCGCAGGTGGTGGTTCTTCG	CGCCGCGAGAGCGTCTCGTGC
cut	X: 7,532,437-7,532,570	GCGCCACCACCCAAAGGTTCACCC	ACAACAGCGCGTGTCAGCTGCCA
Eip75B	3L: 17,985,690-17,985,807	TGTGCCCAGGACCGCGATGTTGT	TGCCAGCAACAGGCCCTTGCCC
BrC	X: 1,515,021-1,515,108	CCGCGAGGGGCGGCAAACAAGTT	TGTGCGTGTGTAGGTTGCCCGATCG
